# Ancient *Borrelia* genomes document the evolutionary history of louse-borne relapsing fever

**DOI:** 10.1126/science.adr2147

**Published:** 2025-05-22

**Authors:** Pooja Swali, Thomas Booth, Cedric C.S. Tan, Jesse McCabe, Kyriaki Anastasiadou, Christopher Barrington, Matteo Borrini, Adelle Bricking, Jo Buckberry, Lindsey Büster, Rea Carlin, Alexandre Gilardet, Isabelle Glocke, Joel D. Irish, Monica Kelly, Megan King, Fiona Petchey, Jessica Peto, Marina Silva, Leo Speidel, Frankie Tait, Adelina Teoaca, Satu Valoriani, Mia Williams, Richard Madgwick, Graham Mullan, Linda Wilson, Kevin Cootes, Ian Armit, Maximiliano G. Gutierrez, Lucy van Dorp, Pontus Skoglund

**Affiliations:** 1 UCL Genetics Institute, Department of Genetics, Evolution & Environment, University College London, London, UK; 2 Ancient Genomics Laboratory, The Francis Crick Institute, London, UK; 3 Bioinformatics and Biostatistics, The Francis Crick Institute, London, UK; 4 Faculty of Science, School of Biological and Environmental Sciences, Liverpool John Moores University, UK; 5 Department of History and Collection Development, Amgueddfa Cymru - Museum Wales, Cardiff, UK; 6 School of Archaeological and Forensic Sciences, University of Bradford, Bradford, UK; 7 Department of Archaeology, University of York, UK; 8 School of Humanities and Educational Studies, Canterbury Christ Church University, Canterbury, UK; 9 Freelance Consultant Osteoarchaeologist, Poulton Research Project, Cheshire, UK; 10 Centre for Palaeogenetics, Stockholm 106 91, Sweden; 11 Environmental Research Institute, School of Science, University of Waikato, New Zealand; 12 Department of Archaeology and history, University of Exeter, UK; 13 iTHEMS, RIKEN, Wako, Japan; 14 Department of Archaeology, University of Reading, UK; 15 Canterbury Archaeological Trust, Canterbury, UK; 16 School of History, Archaeology and Religion, Cardiff University, Cardiff, UK; 17 University of Bristol Spelaeological Society, University of Bristol, Bristol, UK; 18 Honorary Research Fellow, School of Geographical Sciences, University of Bristol; 19 Department of Archaeology, University of York, UK; 20 Host-Pathogen Interactions in Tuberculosis Laboratory, The Francis Crick Institute, London, UK

**Keywords:** infectious disease, genomics, ancient DNA, phylogenetics

## Abstract

Several bacterial pathogens have transitioned from tick-borne to louse-borne transmission, often involving genome reduction and increasing virulence. However, the timing of such transitions remains unclear. We sequenced four ancient *Borrelia recurrentis* genomes, the agent of louse-borne relapsing fever, dating from 2,300 to 600 years ago. We estimate the divergence from its closest tick-borne relative to 6,000–4,000 years ago, suggesting an emergence coinciding with human lifestyle changes such as the advent of wool-based textiles. Pan-genome analysis indicates that much of *B. recurrentis*’ characteristic evolution had occurred by ~2,300 years ago, though further gene turnover, particularly in plasmid partitioning, persisted until ~1,000 years ago. Our findings provide a direct genomic chronology of the evolution of this specialized vector-borne pathogen.

## Introduction

Several species of bacteria have undergone an evolutionary process of transitioning from tick-borne to louse-borne transmission, including the trench fever agent *Bartonella quintana*, the epidemic typhus agent *Rickettsia prowazekii*, and the agent of louse-borne relapsing fever (LBRF) *Borrelia recurrentis*. All species show a pattern of higher virulence in the louse-borne agent compared to their respective closest tick-borne relatives, and all show an evolutionary pattern of genome reduction (1), possibly facilitated by specialisation to the louse vector (1–3). However, the evolutionary time frame and genomic basis of the transition from tick-borne to louse-borne transmission, and the drivers of increased virulence, remain largely unknown.

Relapsing fever, named after the recurring fevers it induces, is caused by several species of *Borrelia*. These are mostly spread by soft-bodied ticks, with the exception of *Borrelia miyamotoi* which is spread by hard-bodied ticks (tick-borne relapsing fever; TBRF) (4) and *B. recurrentis*, which is transmitted from human to human via infected human body louse, *Pediculus humanus (5). Pediculus humanus* is not known to have an animal reservoir and is closely adapted to the human lifestyle. *Borrelia recurrentis* establishes infection when the haemocoel of the infected louse is able to penetrate the mucosa membrane or skin barrier through scratching or infected faeces (6, 7). In contrast to LBRF, most species of TBRF are zoonotic, with multiple animal reservoir hosts, and can be found worldwide.

The present-day *B. recurrentis* genome has an unusual genome structure, comprising a ~930 kb linear chromosome and seven linear plasmids ranging from 6-124 kb in length (8). Though the chromosome is fairly conserved over the *Borrelia* genus, the plasmids have potential to undergo extensive rearrangements (3). *Borrelia recurrentis* has previously been estimated to have lost approximately a fifth of its genome relative to its sister species *B. duttonii*, with prominent gene loss occurring on plasmids (3). It has been suggested that genome loss in the other louse-borne taxa in *Rickettsia* and *Bartonella* was primarily via elimination of inactivated genes (1, 3), which opens up the possibility of a similar process in the evolution of *B. recurrentis*. The exact genes involved in the mechanism for vector specification (louse or tick), and the evolutionary processes underlying genome degradation in *Borrelia*, remain unclear.

Major uncertainties surround the past and present epidemiology of *B. recurrentis* and hence the timeline over which genome reduction and vector/host specialisation occurred. Throughout European history, there have been numerous references to episodes of “epidemic fever”, and “fever lasting six or seven days, with multiple relapses” (9, 10); the earliest descriptions date back to ancient Greece in the 5th century BCE (10). It has been hypothesised that LBRF was the agent of the Yellow Plague which affected Europe in 550 CE, the episodic fevers which became known as sweating sickness in northwestern Europe between 1,485-1,551 CE (11, 12), as well as fevers that accompanied famines in Ireland through the 17th and 18th centuries CE. However, the specific agents of these historical outbreaks have not been confirmed. LBRF posed major challenges to public health during World War I and World War II, before mostly disappearing from Europe at the end of the 20th century CE (13). Today, LBRF remains a major cause of morbidity and mortality in Ethiopia (where it is endemic), Somalia and Sudan (13, 14). While some now consider LBRF as a neglected tropical disease (NTD), LBRF may have the potential to be reintroduced during times of overcrowding, poor access to sanitation and hygiene, and during times of conflict and disaster (3, 6, 15, 16).

*Borrelia recurrentis* is a challenging species to grow in culture, so limited genomic data are available from present-day infections (8, 17). As such, an archaeogenetic approach represents one of the most promising tools for characterising the pathogen’s wider diversity and long-term evolution. Previously, a ~550-year-old (1,430–1,465 cal. CE, 95% confidence) 6.4-fold *B. recurrentis* genome was recovered from a tooth taken from a human skeleton buried in medieval Oslo, Norway (OSL9) (18). However, we lack an understanding of the deeper genomic evolution of *B. recurrentis*, or its prevalence in Europe across time and space. Here we provide four new ancient *B. recurrentis* genomes from Britain spanning 2,300-600 years ago, from the Iron Age to the later medieval period. Leveraging these observations, we confirm the contribution of *B. recurrentis* to disease in European history and document its complex evolutionary behaviour during the transition to louse-borne transmission.

## Results

### Detection and authentication of four ancient *B. recurrentis* genomes

**Figure 1:**
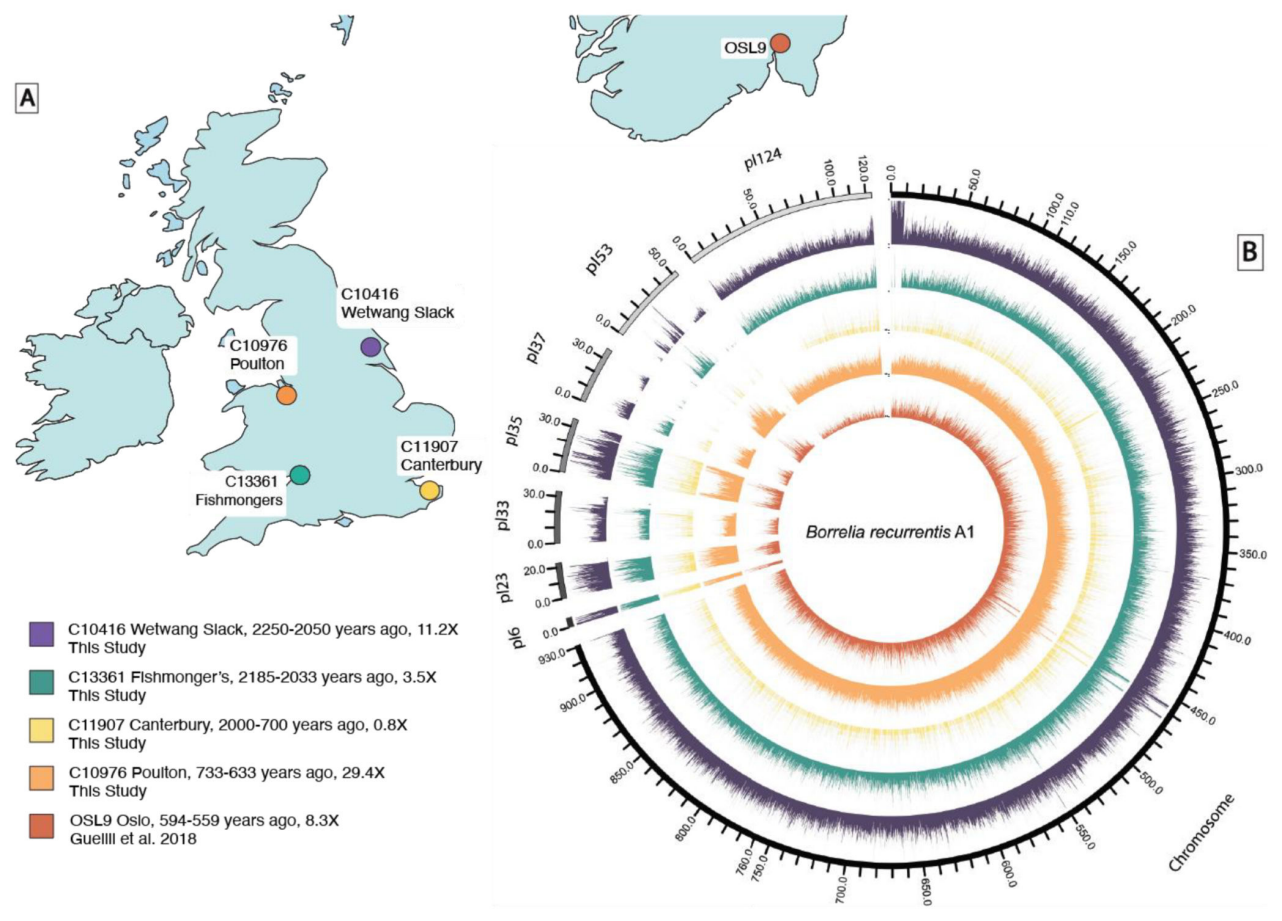
Overview of ancient genomes. A. Geographic location of the four ancient *B. recurrentis* genomes sequenced in this study together with OSL9 previously published by Guellil and colleagues(18). **B**. Circos plot with the coverage of ancient genomes across the *B. recurrentis* chromosome and plasmids when aligned to the *B. recurrentis* A1 reference genome (GCF_000019705.1). A window size of 100bp for the chromosome and 10bp for the plasmids was used to provide the normalised coverage per window plotted. To allow for visualisation, the coverage for each genome was scaled by the maximum coverage per genome (C10416 Wetwang Slack, 70; C13361 Fishmonger’s, 20; C11907 Canterbury, 10; C10976 Poulton, 170; OSL9, 40).

We used an ancient DNA approach, including single-stranded DNA library preparation (19), which optimises retrieval of short fragments and allows postmortem cytosine deamination-derived errors to be removed *in-silico*, to generate whole *B. recurrentis* genomes from four human skeletons recovered from four archaeological sites in Britain (Figure 1A). We generated ~0.8–8.5 billion read pairs per sample, obtaining 0.8–29.4-fold coverage over the *B. recurrentis* A1 reference chromosome (Table 1). All libraries were sequenced to either more than 20-fold coverage or more than 40% clonality (the proportion of sequences identified as PCR duplicates, indicative of library saturation). These include two observations dating to the Iron Age ~2,000 years ago: an 11.2-fold genome (C10416, Burial 240) from the ‘Arras-Culture’-associated Iron Age cemetery at Wetwang Slack, East Yorkshire, contextually dated to 2,300-2,100 years ago (300-100 BCE) (20), and a 3.5-fold *B. recurrentis* genome from Fishmonger’s Swallet (C13361, mandible G10-1.4), a cave in South Gloucestershire, UK. This latter individual has been directly radiocarbon dated to 2,185-2,033 years ago (162 cal BCE-10 CE; 95.4% confidence; 2063±28 BP, BRAMS-5059) (21). We also generated a 0.8-fold *B. recurrentis* genome from the tooth of a cranium (CW29) directly radiocarbon dated to 736-563 years ago (1288-1461 cal. CE; 95.4% confidence with marine correction, 716±25 BP, BRAMS-7370). The cranium probably comes from the Lay cemetery of the late medieval Augustinian friary in Canterbury(22) (founded 1324 CE; (22); see Supplementary Materials (23)). Finally, we generated a 29.4-fold *B. recurrentis* genome (C10976, Sk 435) from a rural cemetery site associated with a medieval chapel next to the village of Poulton, near Chester in Cheshire. The skeleton has been radiocarbon dated to 733-633 years ago (95% confidence, 1,290-1,390 cal CE, 646±14 BP, Wk 52986 (24). Further details of all individuals sampled in this study are available in the Supplementary Materials (23).

Aligned sequences were confirmed to be authentic through assessment of evidence for cytosine deamination (25, 26), distribution of number of mismatches across sequences (edit distance), even coverage across the genome, and a unimodal fragment length distribution (Figure 1B and Figure S1) (27). Additionally, all identified genomes were aligned to representatives of the more closely related species *B. duttonii* Ly and *Borrelia crocidurae* DOU strains, to confirm that all identified cases were genetically closer to the modern-day *B. recurrentis* A1 genome than other related *Borrelia* species (Figure S2). Mapping was also conducted to the wider *Borrelia* plasmid complement.

### Iron Age and medieval lineages of *B. recurrentis*

To evaluate the relatedness of our ancient strains to contemporary sampled strains, we initially reconstructed a *Borrelia* phylogeny including the closest *B. duttonii* representative (Ly). Consistent with the assessment of our samples being *B. recurrentis*, all ancient genomes form a monophyletic clade together with present-day *B. recurrentis* (Figure S3 and Figure S4). Our medieval genome, C10976 Poulton, is positioned in a subclade with the previously published medieval genome from Norway OSL9 (18). Both Iron Age genomes from Britain fall basal to this clade. Among the Iron Age genomes, C13361 Fishmonger’s falls on a lineage basal to C10416 Wetwang Slack, despite being dated to a similar period. Although the Fishmonger’s genome is of lower coverage (2.6-fold when aligned to the core genome), the 100% bootstrap support for this phylogenetic placement suggests the possibility of synchronic sister lineages of the species existing in Britain ~2,300-2,000 years ago. Additionally, we reconstructed a phylogeny on an alignment built using relaxed SNP filtering thresholds in order to include the lower coverage (0.8-fold) C11907 Canterbury genome (23)(Figure S4). Perhaps surprisingly, we found this late medieval Canterbury genome was closely related to Iron Age genomes C10416 Wetwang Slack and C13361 Fishmonger’s which suggests that this clade persisted in Britain for at least 1,400 years. C11907 Canterbury was then excluded from further analysis due to its lower coverage (0.8-fold) and the full extent to which genetic elements from the Iron Age lineages persisted is unknown.

We next reconstructed a core gene alignment to assess the extent to which recombination and accessory (plasmid) gene content may influence our reconstructed relationships, by identifying a set of genes shared amongst the modern sampled diversity of *B. recurrentis*. To do so, we applied the pan-genome analysis tool *Panaroo* (28) to all modern *B. recurrentis* (seven genomes), *B. duttonii* (two assemblies) and *B. crocidurae* (two assemblies) genomes (Table S1). The inferred *Borrelia* pan-genome comprised a total of 3,035 genes after removing pseudogenes and genes of unusual length, corresponding to a length of 2,223,831 base pairs. We observed a high degree of conservation within the *B. recurrentis* species, supporting a limited intraspecies pan-genome despite high plasmid carriage. Of these genes, we identified 933 as being present in 99% of included strains, providing a core gene reference panel to which the ancient samples were aligned before phylogenetic reconstruction. Phylogenies constructed by mapping to the *B. recurrentis* A1 reference genome and core-genome alignment showed identical phylogenetic topologies as well as a similar number of SNPs using both approaches (4,192 SNPs versus 4,200 SNPs) albeit with improved bootstrap support values in the latter. This suggests a limited impact of mapping bias or structural variation on our observed patterns of relatedness.

Given that recombination may violate the assumptions of tree-building representations of diversity, we formally tested for evidence of homologous recombination using ClonalFrameML (29). We observed very limited evidence of homologous recombination, with only a small fraction of the genome (<0.1%) estimated to derive from such processes. Nonetheless, after pruning the alignment for the modest amount of recombination detected by ClonalFrameML, we recovered a topologically identical phylogeny (Figure S5).

### Chronology of the divergence from the tick-borne sister species

The timeline over which *B. recurrentis* diverged from its common ancestor *B. duttonii*, and subsequently evolved a different arthropod vector transmission route, is uncertain. Here, we use our ancient *Borrelia* time series to calibrate the joint genealogical history of modern and ancient *B. recurrentis* and its mutation rate. We first tested a hypothesis of clock-like evolution in our core-genome alignment. We formally assessed the temporal structure in our recovered phylogenies using *BactDating* (30) to test for a significant correlation between genomic diversity and sampling time using date randomisation (23). We assessed temporality including and excluding C13361 Fishmonger’s, due to its lower coverage, and obtained an R^2^ of 0.69 (p-value 0.00080) and 0.66 (p-value 0.0011) respectively. This result suggests a significant temporal signal across our dataset (Table S2).

**Figure 2:**
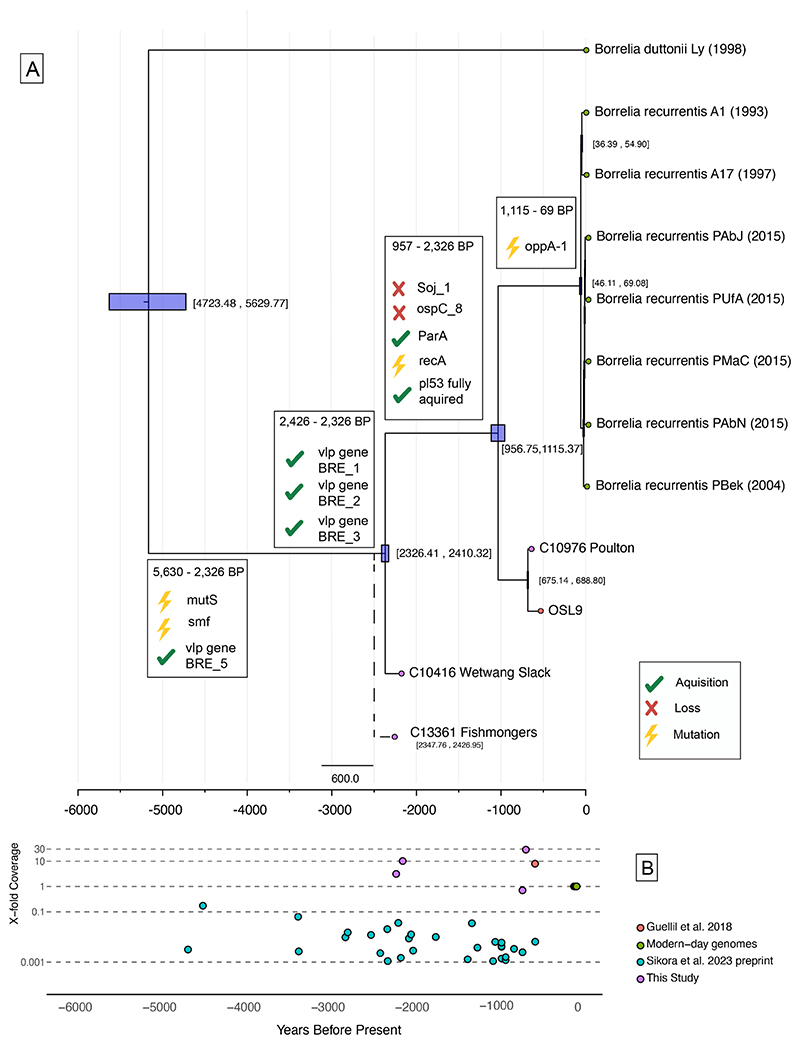
Temporal evolution of *Borrelia recurrentis*. A. Bayesian tip-calibrated maximum clade credibility time tree from Beast2, providing the best supported model following path-sampling. The 95% highest posterior density is indicated with purple boxes and within brackets. The placement of Fishmonger’s is indicative following a relaxed tip-calibration analysis. Ancient samples are highlighted by coloured tips. Key gene acquisition and loss events described in the text are highlighted at the relevant phylogenetic nodes. **B**. Timeline providing the estimated age and X-fold coverage (on a log10-scale) of *B. recurrentis* observations recovered from ancient DNA in this and other studies.

We next implemented formal Bayesian tip-dating calibration via *BEAST2* (31) to provide a probabilistic assessment of the divergence of sampled *B. recurrentis* from the closest sequenced relative *B. duttonii* Ly. This approach jointly estimates the rate of mutation over the non-recombining fraction of the core alignment (23). Evaluating a suite of possible clock and demographic models, we estimate a split time of all ancient *B. recurrentis* genomes with at least 5-fold coverage in our dataset from *B. duttonii* Ly ranging from 2,215-5,630 years ago (95% HPD interval across models). When the lower coverage C13361 Fishmonger’s sample is included, we estimate the split time from *B. duttonii* Ly to between 2,313-7,654 years ago, overlapping with the initial estimate (Figure 2A and Figure S6, Figure S7, Table S3). The best supported model indicates a divergence estimate of 5,156 (95% HPD 4,724-5,630) years ago, corresponding to a rate of evolution of 5.0 x 10^-8^ (4.6 x 10^-8^ - 5.5 x 10^-8^ substitutions per site per year; 95% HPD values) (Figure 2A). Estimates from this model suggest an emergence of the Iron Age clade between 2,326-2,410 years ago with the medieval clade, including C10976 Poulton and the previously recovered OSL9 genome, dating to within the last 700 years. Our inference would also suggest a very recent emergence (46-69 years ago) of all contemporarily sampled *B. recurrentis* infections (which are exclusively from Africa or linked to refugee status), with a caveat that both ancient and modern diversity is significantly undersampled.

To validate the Bayesian inference of a relatively recent divergence of *B. recurrentis* from the shared ancestor with *B. duttonii* Ly, we also performed an additional analysis. Here, we identified SNPs where *B. crocidurae* DOU and *B. duttonii* Ly both had an alternative variant to all modern *B. recurrentis* genomes, which we can interpret as new mutations occurring on the lineage leading to the modern *B. recurrentis* clade since the divergence (23). This approach also has the advantage of excluding any impact of sequence errors specific to the ancient genomes. If divergence occurred approximately 5,000 years ago, we could expect the ancient individuals to have accumulated these mutations in an approximately clock-like manner, with e.g. the ~700-year old medieval genome, C10976 Poulton, having at most 1-(700/5000)=86% of these derived mutations, and an Iron Age ~2,200-year-old genome having at most 57%. Indeed, these expectations match what we observe in the empirical data (Table S4), and we find an intercept in linear regression of ~6,100 years ago, overlapping with our estimates following Bayesian tip-dating calibration when C13361 Fishmonger’s is included. We note that this number is expected to slightly overestimate the true divergence because the TMRCA of *B. recurrentis* A1 and the ancient genome will always be slightly older than the age of the sample. A divergence date from *B. duttonii* Ly in the Late Neolithic implicated in our temporal analysis is further supported by Sikora *et al*’s preprint study providing low-coverage observations of *B. recurrentis* up until ~4,600 years ago, with the earliest observation identified in a Late Neolithic individual from Denmark (32) (Figure 2B).

### Patterns of pan-genome diversity and genome reduction in *B. recurrentis*

To assess previously suggested genome reduction in *B. recurrentis*, we aligned each of our ancient observations and all modern representatives to both of the *B. duttonii* Ly and *B. duttonii* CR2A genomes and assessed the fraction of genome covered. With such an approach, simulated aDNA-like sequences from modern *B. recurrentis* successfully map to ~98.6% of the *B. duttonii* Ly chromosome (Table S5), though with as much as ~20% of the whole *B. duttonii* Ly genome being absent in *B. recurrenti*s. The two higher-quality ancient genomes from medieval and Iron Age lineages both showed highly similar numbers of 98.9% to 98.6% chromosomal coverage. While we note marked variability in the accessory genome, the chromosomal coverage supports conservation in similarity to *B. duttonii* Ly between *B. recurrentis* ~2,300 years ago and samples from contemporary infections.

**Figure 3:**
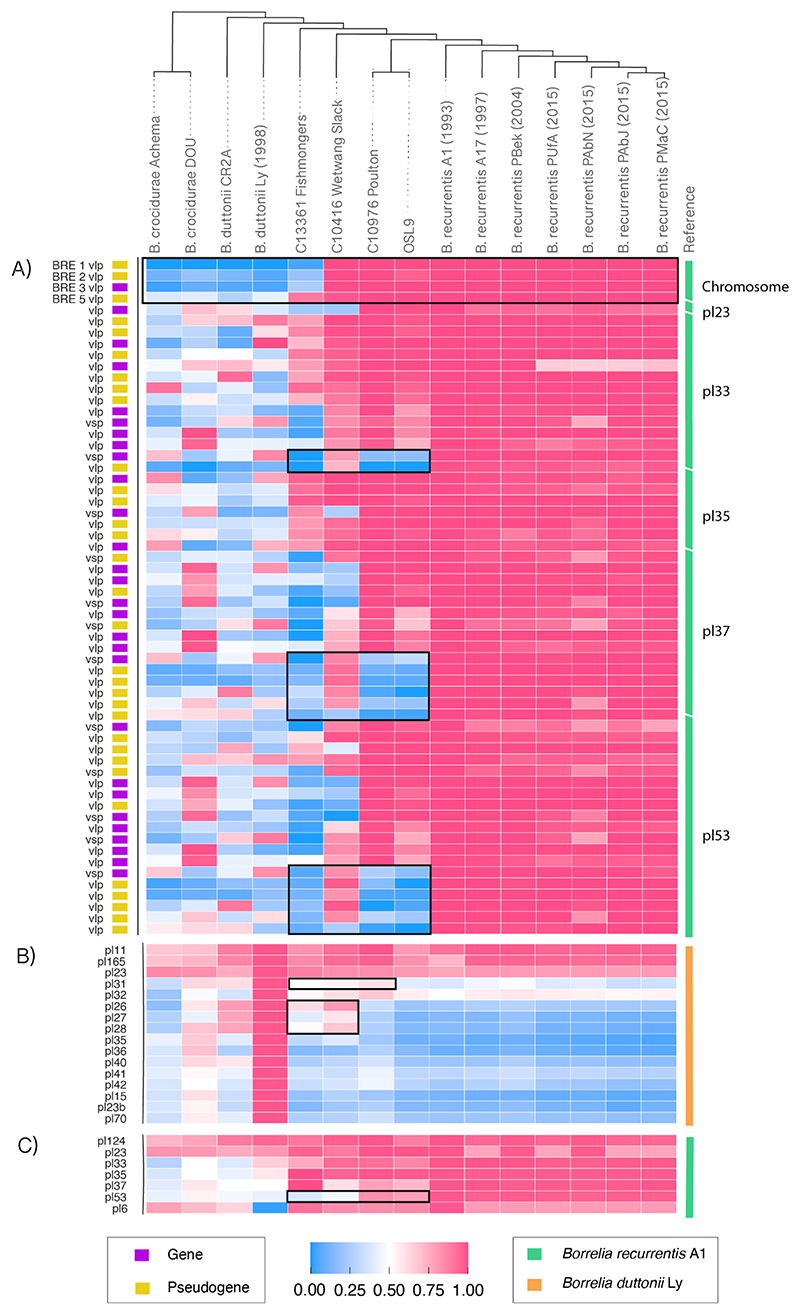
Gene losses/gains across variable major proteins (vmp), *B. duttonii* Ly and *B. recurrentis A1* plasmids. Ancient and modern genomes were aligned to single reference *B. recurrentis* (green) or *B. duttonii* (orange) (23). Regions of interest highlighted in the text are outlined with a black box. Cladogram provides the relationship between different genomes based on a SNP phylogeny. **A)** Normalised breadth of coverage across the variable major proteins on the *B. recurrentis* A1 chromosome and plasmids (pl) (Table S8), using *BEDTools* v2.29.2. Coordinates of the vmp genes and whether they are classified as genes (yellow) or pseudogenes (purple) were provided in Guellil *et al*. (18) using previously annotated genomes from the NCBI database. **B)** Breadth of coverage for *B. duttonii* Ly plasmids using SAMTools v1.3.1 with a mapping quality of Q1. **C)** Breadth of coverage for *B. recurrentis* A1 plasmids using *SAMTools v1.3.1* with a mapping quality of Q1.

We next assessed the contribution of plasmid carriage relative to *B. duttonii, B. crocidurae* and contemporary *B. recurrentis* (Figure 3). We observe a diversity in plasmid carriage across the clade when aligned to *B. duttonii* Ly. For instance we detect 10 cases (plasmids pl15, pl23b, pl35, pl36, pl40, pl41, pl42, pl70, pl31, pl32) where coverage over the plasmid is seen at <75% in all closely related species (Table S5). We identify three plasmids (pl26, pl27, pl28), in *B. duttonii* Ly(3, 33), that are present in the Iron Age genomes—authenticated using coverage, cytosine deamination patterns and distribution of mismatches—but are absent or at substantially lower coverage in medieval and present-day genomes (Figure S8, Table S5). We therefore suggest at least partial plasmid loss events, or loss of significant plasmid-borne elements, ~2,000-700 years ago, between the Iron Age and medieval lineages. Overall, by the medieval period, *B. recurrentis* harboured the full suite of plasmids observed in currently sampled infections. In contrast, the Iron Age genome shows only partial coverage (~40%) of *B. recurrentis* plasmid pl53, which is present in medieval and modern genomes, suggesting that the complete plasmid gene complement was acquired after ~2,000 years ago (Table 1, Figure 3).

We used a pan-genome framework to establish patterns of gene content across *Borrelia* (23). Such an approach is agnostic to chromosomal or plasmid affiliation, where our ancient *B. recurrentis* genomes may represent possible intermediates on the trajectory towards specialisation. First, we assessed the full gene repertoire across all *B. crocidurae, B. duttonii* and *B. recurrentis*. A total of 3,184 unique genes were identified, defining the pan-genome (23). We recover a total pan-genome size by species of 1389-1701 (*B. crocidurae*), 1544-1626 (*B. duttonii*) and 1149-1170 (*B. recurrentis*) genes respectively, suggesting *B. recurrentis* carries a gene repertoire ~25% of the total gene count of its closest relative (Figure S9). Notably the gene content by species includes a number of unique genes, including 165 in *B. recurrentis*, highlighting the dynamic nature of the accessory gene component likely as a result of the extensive plasmid carriage in *Borrelia*. Intersecting the presence of genes in the three species, we find that 25 genes are absent in *B. recurrentis* A1 but present in all four genomes representing the other two species, as opposed to 18 genes absent in both *B. duttonii* genomes but present in the genomes from the other two species. We further applied an ancestral state reconstruction approach (23) to reconstruct the ancestral accessory genome in each case, though noting limited sampling of contemporary species. In doing so we recover 92 accessory genes which are estimated to have been present in the ancestor of *B. crocidurae* and *B. duttonii* but not *B. recurrentis* (Table S6). The pan-genome was then filtered for fragmented genes and or pseudogenes resulting in 3,035 genes (28). Ancient and modern genomes were then aligned to this pan-genome and the normalised coverage across these genes were assessed (23). The normalised coverage was then subjected to presence/absence assessment, filtering and annotation, resulting in 71 genes which showed temporal patterning (Table S7, Figure S10).

Given hypotheses surrounding genome reduction and plasmid stability, we particularly noted two genes in the pan-genome implicated in plasmid segregation and partitioning, *Soj* and *ParA* (annotated as *Soj_1* and *ParA_1*), which showed temporal patterning across our dataset (Table S7, Figure S10). *Soj_1* is present in both *B. crocidurae* and *B. duttonii*, and the ancient Iron Age *B. recurrentis* genomes of sufficient coverage for consideration, but absent in medieval and present-day data. Assuming parsimony, this suggests gene loss on the branch leading to the descendant medieval and contemporary clades (we estimate phylogenetically, based on current data, this occurred between 2,326-1,115 years ago; 95% HPD). In contrast, *ParA*, an ortholog of the *Soj* gene(34) and also implicated in plasmid segregation, is present solely in the medieval and modern-day genomes. A parsimonious explanation is the acquisition of this gene between the Iron Age and the medieval period. Exploration of the genomic neighbourhoods of both the *ParA_1* and *Soj_1* genes (23) further supports that these genes are today localised on different backgrounds, with *Soj_1* found on the *B. duttonii* Ly chromosome and *ParA_1* on the pl53 *B. recurrentis* plasmid, which as previously mentioned, is only partially observed in the Iron Age genomes.

### Temporal variation in functional genes

*Borrelia* relapsing fevers use a suite of predominantly plasmid-encoded antigenic phase variation as a mechanism of immune evasion, though we observe four of these genes at the beginning of the *B. recurrentis* A1 chromosome, which have been uniquely acquired (3). Immune evasion is mediated by variable large proteins (vlp) and variable short proteins (vsp), together known as the variable major proteins (vmp). Antigenic variation of these surface-exposed lipoproteins likely plays an important role in evading host-acquired immunity, allowing for the bacteria to persist within its host population. However, it is unclear how stable this mechanism has been through evolutionary time (35–37). Of the chromosomal vmp genes, we found that the medieval lineage, comprising C10976 Poulton and OSL9, had similar vmp profiles, both to each other and to present-day *B. recurrentis*, with all four chromosomal-borne vmps characteristic of present-day *B. recurrenti*s having been gained by medieval times (Figure 3, Table S8). However, two of these vlp genes have been identified as pseudogenes in modern *B. recurrentis* genomes (3), and so, despite having been gained by the time of the *B. recurrentis* Iron Age genomes, these are believed to be functionally redundant in modern genomes.

When evaluating vmp profiles over the *B. recurrentis* plasmids, we noted the absence of a number of vmp genes in the medieval genome, many of which are pseudogenes. This was seen in particular at the 3’ ends of the pl33, pl37 and pl53 plasmids (Figure 3); an observation also seen in the previously published OSL9 genome from the same time period (18). Interestingly, these regions (3’ pl33, pl37 and pl53) are mostly present in the Iron Age sample C10416 Wetwang Slack but are absent in our basal Iron Age sample C13361 Fishmonger’s. Due to the lower overall coverage of the Fishmonger’s genome, the exact gene complement of plasmid-borne vmp genes is difficult to formally assess. However, this observation is consistent with the potential for interspecies and intraspecies variability in vmp profiles (Table S8), as is seen in its tick-borne relatives (*B. crocidurae* and *B. duttonii*).

Evaluating other hallmarks of infective behaviour (3), we note that BDU 1, a p35-like antigen implicated in fibronectin binding in *Borrelia burgdorferi* (Lyme disease) (3, 38), is absent in *B. recurrentis*. This outer membrane protein is also absent across our Iron Age and medieval observations supporting an early loss following the split with *B. duttonii Ly (*Figure S10). A similar pattern was seen for several further outer membrane proteins, including those involved in host complement system inactivation (BDU 2, BDU 3, BDU 5)(36), which are absent in *B. recurrentis* from ~2,000 years ago by our earliest observation. Other genes implicated in evading host innate defences (*cihC* and *fhbA*) were however maintained. More globally, we observe some temporal variation which suggests an ongoing process of genome adaptation, for example, the aforementioned loss of *Soj* (annotated as BDU 429 on the *B. duttonii* Ly reference) (Figure S10) and truncation of the uncharacterised protein, BDU 430, in the modern *B. recurrentis* genome. These genes are also absent/truncated in the medieval genomes, but are present in genomes from the Iron Age period.

Finally, we assessed SNPs and indels of functional relevance (Figure S11) (3). For example, the *oppA-1* gene is a pseudogene in *B. recurrentis* A1 due to an in-frame stop mutation; but in *B. burgdorferi*, it is shown to play an essential role in metabolic function as well as survival in different environments (39). As with the previously reported medieval genome from OSL9 (18), this gene is found in its ancestral form in all of our ancient samples. This suggests the inactivation of *oppA-1* occurred relatively recently, we estimate within the last ~1,115 years (Figure 2). Similarly, the *smf* gene and the *mutS* gene show an in-frame stop mutation and a frameshift mutation in present-day *B. recurrentis*. All ancient genomes with data at this locus show the in-frame stop mutation in the *smf* gene, with the higher coverage genomes (C10416 Wetwang Slack and C10976 Poulton) also supporting the presence of the *mutS* frameshift mutation. Conversely, the *recA* gene is still functional in the high-coverage Iron Age genomes. Hence, the true recombination efficiency is unknown for these genomes even if we find little detectable signal of recombination in our genomics analysis (Figure S5).

## Discussion

Here we reconstruct the complex evolutionary history of *B. recurrentis* by retrieving and analysing four ancient *Borrelia* genomes from Britain across a ~1,500-year time span. Our work confirms the presence of the pathogen in Europe during both the Iron Age and the later medieval periods, extending the high-coverage *B. recurrentis* whole-genomes by approximately 1,600 years. This detection adds markedly to existing data from the species, with only eight genomes (seven contemporary and one medieval) available prior to this study. While it is unclear whether there is any link between our detected infections and historically-attested outbreaks in Britain, we note that the high coverage recovery of genomes achieved here suggests that the individuals studied likely died from acute infections with high levels of bacteremia. The likely high false negative rates of infectious disease detection in ancient remains means that it is not possible to link these results with absolute rates of disease amongst past populations. Without antibiotic treatment LBRF infections are fatal in 10-40% of cases (40); however, it is uncertain to what extent this figure would be applicable to ancient cases of the same disease, which differed genetically from modern forms and operated in different cultural and environmental contexts.

Aided by our new genomic observations, our work confirms the existence of a closely related medieval phylogenetic clade of *B. recurrentis* that existed from at least 600 years ago and spanned Britain and the Scandinavian peninsula. In addition, we recover previously unknown ~2,000-year-old basal lineages from Iron Age Britain. The phylogenetic placements of the Iron Age genomes may suggest that multiple lineages of *B. recurrentis* existed at this time. The clustering of the low-coverage C11907 Canterbury genome with this clade, further implies that this lineage persisted in Britain for at least another ~1,400 years, extending into the medieval period.

Harnessing this temporal structure, we find support for a relatively recent divergence of sampled *B. recurrentis* strains from the closest relative *B. duttonii* Ly 5,600-4,700 years ago. While we caution that a more complete time series could improve these calibrations and divergence estimates, the evidence is consistent with a Late Neolithic/Early Bronze Age emergence of the agent of LBRF. Given the human host-specificity of *P. humanus*, it is notable that this emergence time coincides with changes in human lifestyles, potentially resulting in the human body louse becoming a more favourable vector. Good examples of this would be the gradually increasing levels of sedentism and contact with domesticates during the ongoing development of agriculture and pastoralism, as well as the emergence of densely-occupied settlements in regions of Eastern Europe specifically (41). Our estimated divergence also coincides with the development of sheep farming for wool in the Near East, Caucasus and/or Pontic-Caspian steppe from c.6000 years ago, which eventually leads to an extensive western Eurasian wool trade from at least c.4000 years ago (42–44). Compared to plant-based textiles, woollen clothing may have created more favourable living conditions for lice, as rougher material is preferred for egg laying (45). It is plausible that additional temporal resolution may be available by considering the evolution of the human body louse, which is thought to derive from human head lice (46, 47). However, to date the chronology of this niche transition is unclear.

To date, no *B. recurrentis* whole genomes have been identified earlier than our divergence estimates. Sikora *et al*. (32) report 31 observations of *B. recurrentis* in the ancient skeletal record in an unpublished preprint. These findings are based on the assignment of sequencing reads from human skeletons, mostly from Eurasia (Figure 2B and Figure S12), all of which are lower than 0.17-fold genome coverage. The earliest recovered observation is from Denmark dating to ~4,600BP (RISE61; 0.003X) (32, 48). Due to the low coverage of these *B. recurrentis* observations through time, it was not possible to incorporate them into our phylogenetic analysis nor inspect gene content. However, further sequencing of these or other ancient samples will be important in establishing the spatio-temporal timeline of infections, as well as to potentially uncover previously unseen genomic diversity. Similarly, contemporary infections are also undersampled, with very limited available genomic data mostly linked to cases in East Africa or associated with migrants on their journey to Europe (40). As such, it is challenging to disentangle many of our observations in ancient strains from expectations arising from population structure between Africa and Europe. Further data from different time periods and geographic regions of the world may result in our temporal estimates being amended. This would be required to assess any connection between the population structure of the bacteria and the mobility of its human hosts.

Our estimates over the non-recombining proportion of the genome indicate a recent timeline over which presently sampled *B. recurrentis* diverged from its closest sequenced relative, *B. duttonii* Ly. We note, based on Intergenic spacer (IGS) typing, that some have suggested that *B. recurrentis* is a degraded form of *B. duttonii* and hence the lines between species demarcations may well have been blurred in deeper history (49). Aside from undersampling, we must also consider the possibility of rate variation in the history of *B. recurrentis*. While a relaxed clock model was less well supported by our Bayesian phylogenetic analyses, it is plausible that ecological influences on mutation rate, as observed in other bacterial pathogens, may have played a complex role which is difficult to capture using our temporal reconstruction approach (50, 51).

Nonetheless, our work indicates the need for the processes shaping *B. recurrentis* to have occurred within only 5,600-4,700 years since its divergence from *B. duttonii Ly*, with genome reduction linked to specialism towards the human body louse vector and potentially resulting in enhanced pathogenicity (51). We observe that a fraction of the *B. duttonii* genome was already missing by the time of our oldest sampled *B. recurrentis* infection ~2,000 years ago and document a marked reduction in pan-genome size with moderate shared gene loss. This evolution, particularly in light of the slow global mutation rate, may have been supported by plasticity in the wider accessory genome of *Borrelia*. Indeed, our work highlights that gene acquisition may occur during the process of genome decay, with *B. recurrentis* itself harbouring 165 genes otherwise not seen in the wider relapsing fever clade. Similarly, we detect evidence of gene acquisitions in each of the two *B. crocidurae* and *B. duttonii* genome assemblies that are currently available, with within-species variation that is itself likely under-characterised (49). It is also relevant that the species reference genomes available are from in vitro cultivated spirochaetes which may be susceptible to genomic plasticity, for example plasmid loss in culture has previously been documented in related *Borrelia burgdorferi* (52). As such it becomes challenging to precisely estimate the genome size difference between the two species, with further whole genome sequencing required to capture within and between species heterogeneity. Nonetheless, aided by the high coverage Iron Age and medieval *B. recurrentis* genomes, we can demonstrate that at least some of the decay towards the extant *B. recurrentis* genome was ongoing over these periods. In particular, we document the partial loss of three plasmids (or extensive plasmid-borne elements) between *B. duttonii* and our Iron Age samples and later medieval and contemporary *B. recurrentis* strains; estimating that this event most likely occurred between 2,326 and 1,115 years ago.

The extent to which such loss events may still be ongoing is unclear, though it has been suggested that disruption in plasmid partitioning genes relative to *B. duttonii* homologs may indicate a degree of an ongoing reductive process (3). Indeed, we note an interesting temporal pattern of major chromosomal and plasmid partitioning genes *Soj* and *ParA*, best described for their role in *Bacillus subtilis* and *Escherichia coli* (53). While it has previously been reported that *B. recurrentis* lacks a chromosomal *Soj* homologue (3), using a pan-genome approach we identify that *Soj* was retained until at least the Iron Age. The homologue was then lost by the earliest of our two medieval observations, where we simultaneously reconstruct the gain of *ParA*, a distant homolog of *Soj*. Such observations highlight the fluidity of the process of genome reduction, with the suggestion of necessary acquisition of some functionally relevant genes as a likely outcome of large-scale plasmid loss events. Another plausible contributor to the pattern of genome decay is the loss of DNA repair mechanisms, with the inactivation of genes such as *recA* resulting in the bacteria becoming dependent on its human/vector hosts (3, 8, 54). Our data suggest that the loss of *recA* as a DNA repair mechanism may also be a reasonably recent event, given that we find the *recA* gene is still likely functional in our Iron Age observation. It is significant to note that both *smf* and *mutS*, also implicated in DNA repair, are disrupted across our ancient samples, supporting the importance of this mechanism in the wider propensity for genome loss.

The transition to a host-specialised pathogen from ancestral groups exhibiting a broader host-range will also have exerted a selective pressure on the bacteria. Within *Borrelia*, the vmp and vmp-like genes offer an important mechanism to allow persistence and resurgence of relapsing fevers, with antigenic variation during infection through a process of silent vmp genes being transferred to the expression locus, leading to the generation of new surface protein variants (35–37). Our work supports some temporal variability within *B. recurrentis*, particularly in the vmp genes located at the 3’ end of the pl33, pl37 and pl53 plasmids, which we observe as absent in medieval samples. The downsizing of the vmp repertoire may act to modulate antigenic behaviour, though likely reflects the loss of genes during adaptation to a more specialised vector host relationship. It is also plausible that the pl33, pl37 and pl53 plasmids are shorter or subject to genomic rearrangements in these strains. This observation was also suggested by Guellil and colleagues who detected a similar patterning in the only other ancient *B. recurrentis* full genome published prior to this study (18). We also note far richer diversity in vmp profiles in non-*recurrentis* species, indicating that the extent of antigenic plasticity may have been very different prior to host specialisation. Though the antigenic variation of the vmp genes is the best-known mechanism to evade host immunity, we also note retention of genes involved in C4b-Binding and the Factor-H binding proteins in both *B. recurrentis* and *B. duttonii* (55).

Together we highlight how ancient microbial DNA can be used to enhance our understanding of the age and diversity of significant but understudied pathogens. Our work highlights the value of temporal data in pinpointing the timing and patterning of the process of host/vector specialisation, supporting a prevailing background of genome reduction, notwithstanding more recent key instances of gene gains and losses. While we cannot strictly exclude that these ancient bacteria from Britain were tick-borne, genomic features such as a similar observed genome evolution seen in present-day louse-borne *Borrelia*, and given the known geographic range of relevant tick species today, certainly makes it most parsimonious that they were adapted to be a louse-borne form of relapsing fever. Additional work is required to build a mechanistic understanding of the genomic basis for each vector niche.

## Material and Methods Summary

*Borrelia recurrentis* infections were identified in four individuals each from different archaeological sites across Britain dating to the Iron Age (Wetwang Slack and Fishmonger’s Swallet) and medieval period (Canterbury and Poulton). Approximately 11-35 mg of dentine powder from each individual was sampled and underwent single-stranded library construction and whole-genome shotgun sequencing. Upon detection of *B. recurrentis*, via *kraken2* (56), libraries were further sequenced to ~0.8–8.5 billion read pairs per sample, obtaining 0.8–29.4-fold coverage over the *B. recurrentis* A1 reference genome. Given the poor availability and diversity of contemporary genomes, we utilize the diversity from closely related species, *B. duttonii* and *B. crocidurae* as well as all available *B. recurrentis* isolates in our analyses. Firstly, we reconstructed a core gene alignment to assess the extent to which recombination and accessory (plasmid) gene content may influence our reconstructed relationships. To do so, we applied the pan-genome analysis tool *Panaroo* (28) and identified a set of core genes shared amongst these contemporary isolates and extracted a core gene reference which we aligned our ancient and modern genome to. We then reconstructed relatedness using a maximum likelihood approach in IQ-TREE v.1.6.12. We pruned the alignment for evidence of recombination using ClonalFrameML (29) and assessed the temporal signal of our dataset using *BactDating* (30). Given strong temporality in our dataset, we implemented formal Bayesian tip-dating calibration via *BEAST2* (31) to provide a probabilistic assessment of the divergence of sampled *B. recurrentis* from the closest sequenced relative *B. duttonii* Ly and simultaneously estimated the mutation rate across the recombination pruned core genome. To reconstruct gene gains and losses across the pan-genome, we used a dataset of contemporary isolates to identify 3,035 genes across *B. recurrentis* and closely related species. Simulated aDNA-like modern and ancient sequences were mapped back to all identified genes and their normalised coverage was assessed. Genes that showed a temporal patterning were further investigated and coupled with the time tree from *BEAST2*, the occurrence of the gain/loss event was temporally estimated. Additionally, a standard reference-based mapping approach to both *B. recurrentis* A1 and *B. duttonii* Ly was used to corroborate these evolutionary events, manually inspect SNPs/Indels of interest and assess the *vmp* carriage across the ancient genomes. Finally, to investigate the process of genome reduction, we mapped our simulated aDNA-like sequences from modern *B. recurrentis* genomes, as well as our ancient genomes, to the *B. duttonii* Ly chromosome and plasmids, assessing their coverage. To evaluate to what extent unique gains in *B. duttonii* Ly could inflate the estimates of genome reduction in *B. recurrentis*, we used an ancestral state reconstruction approach to reconstruct the ancestral accessory genome for the modern isolates.

## Supplementary Material

Figure S1

Table S1

## Figures and Tables

**Table 1:** Sequencing metrics for the four individuals recovered in this study when mapped to the *B. recurrentis* A1 reference genome (chromosome and plasmids) requiring a mapping quality of MQ1.

Individual		C10416 Wetwang Slack (Burial 240)	C10976 Poulton (Sk 435)	C13361 Fishmonger ’s (G10-1.4)	C11907 Canterbury (CW29)
**Archaeological dates (years ago)**		2,250-2,050 (context)	733-633 (C14 dating)	2,185-2033 (C14 dating)	736-563 (C14 dating)
**Total sequences generated after adapter merging**		8,453,668,8 64	841,565,272	2,615,551,7 50	2,040,000,5 13
**Sequences aligned to *B. recurrentis* A1 (Chromosome and accessory genome)**		526,415	888,736	139,860	164,451
**Sequences aligned after duplicate removal**		264,871	715,574	82,053	18,372
**Clonality (%)**		49.7	19.5	41.3	88.8
**Proportion *B. recurrentis* (%)**		0.0062	0.1056	0.0053	0.0081
**X-fold coverage when aligned to *B. recurrentis* A1 chromosome and plasmids (overall Q1) [all]**		10.2	28.6	3.1	0.7
**Breadth of coverage >1x (%) [all]**		95.6	98.7	85.2	37.1
**Breadth of coverage >2x (%) [all]**		93.1	98.6	65.9	13.0
**Breadth of coverage >3x (%) [all]**		89.3	98.4	44.5	4.6
**Chromosome (NC_011244.1)**	**X-fold coverage**	11.2	29.4	3.5	0.8
	**Breadth of coverage >1x (%)**	99.6	100.0	90.4	40.3
**Plasmid pl6 (NC_011263.1)**	**X-fold coverage**	18.8	57.4	6.5	2.1
	**Breadth of coverage >1x (%)**	85.9	86.1	85.4	65.0
**Plasmid pl23 (NC_011252.1)**	**X-fold coverage**	13.7	48.2	4.2	1.0
	**Breadth of coverage >1x (%)**	90.6	99.7	87.6	52.7
**Plasmid pl33 (NC_011253.1)**	**X-fold coverage**	6.6	16.5	1.7	0.6
	**Breadth of coverage >1x (%)**	95.1	94.4	69.3	31.9
**Plasmid pl35 (NC_011255.1)**	**X-fold coverage**	13.1	40.9	3.7	1.1
	**Breadth of coverage >1x (%)**	96.9	100.0	92.5	55.2
**Plasmid pl37 (NC_011258.1)**	**X-fold coverage**	3.1	14.8	0.7	0.2
	**Breadth of coverage >1x (%)**	61.1	80.4	34.8	14.7
**Plasmid pl53 (NC_011260.1)**	**X-fold coverage**	2.9	22.5	0.9	0.2
	**Breadth of coverage >1x (%)**	45.3	86.1	34.1	9.0
**Plasmid pl124 (NC_011246.1)**	**X-fold coverage**	6.5	23.4	2.0	0.3
	**Breadth of coverage >1x (%)**	98.0	100.0	82.9	22.8
**To Core Genome Alignment**	**X-fold coverage**	9.4	27.4	2.6	0.5
	**Breadth of coverage >1x (%**	96.9	98.0	87.2	36.7

## Data Availability

All sequence data is available in the European Nucleotide Archive with accession number PRJEB82956.
