# Supplementary material for: Ancient Borrelia genomes document the evolutionary history of louse-borne relapsing fever
Source: Science. Author manuscript; Available in PMC 2025 Jun 24. (PMC7617810; doi:10.1126/science.adr2147)
Supplement: Figure S1 [file EMS206317-supplement-Figure_S1.pdf]

Science
AAAS

Supplementary Materials for

Ancient *Borrelia* genomes document the evolutionary history of louse-borne relapsing fever

Pooja Swali *et al*.

Corresponding authors: Pooja Swali, pooja.swali.18@ucl.ac.uk; Lucy van Dorp, lucy.dorp.12@ucl.ac.uk; Pontus Skoglund, pontus.skoglund@crick.ac.uk

Science **388**, eadr2147 (2025)
DOI: 10.1126/science.adr2147

The PDF file includes:

Materials and Methods
Figs. S1 to S15
References

Other Supplementary Material for this manuscript includes the following:

MDAR Reproducibility Checklist
Tables S1 to S11

Materials and Methods

Archaeological Context:

Summary: Wetwang Slack is an Iron Age Arras-style cemetery in East Yorkshire dating to 2,300-2,100 years ago (300-100 BCE). Sample C10416 was taken from a left mandibular third molar from Burial 240. Fishmonger's Swallet is a cave site in South Gloucestershire. Sample C13361 was taken from a left first molar in a disarticulated human mandible (G10-1.4) that has been radiocarbon dated to 2185-2033 years ago (162 cal. BCE - 10 cal. CE; 2063±28 BP, BRAMS-5059 (*21*)). Sample C11907 was taken from a maxillary left third molar from an ancient cranium (CW29) that has been radiocarbon dated to 736-563 years ago (1288-1461 cal. CE with marine correction, 716±25 BP, BRAMS-7370), and while the precise provenance is uncertain, it is likely to come from the late medieval Lay cemetery associated with the Augustinian Friary in Canterbury. Sample C10976 was taken from a maxillary canine of an adult male (Sk 435) buried in a cemetery associated with a medieval chapel at Poulton, Cheshire. Radiocarbon dates from Sk 435 and other parts of the cemetery indicate that Sk 435 dates to 733-633 years ago (1290-1390 cal. CE, 646±14 BP, Wk 52986 (*24*)).

Wetwang Slack: The individual with LBRF is Burial 240 at Wetwang Slack, a square-ditched barrow cemetery in East Yorkshire associated with the 'Arras Culture' dating to 300-100 BCE (*57, 58*). The human remains are curated at the University of Bradford on loan from the Hull and East Riding Museum. This individual, identified osteologically as an adult female, was buried without any accompanying grave goods under a square barrow. The lack of grave goods is not especially unusual at Wetwang Slack, even for primary barrow burials such as this. This individual has been identified as the mother or daughter (order currently unknown) of another adult female (Burial 303), and a second-degree relative of a third adult female (Burial 270), both also primary burials under square barrows. This suggests that she was likely a locally-born member of the community and, given that she was a primary rather than secondary burial, she was probably of reasonably high status (although not in any major hierarchical sense given the numbers of barrows present at Wetwang Slack).

Fishmonger's Swallet: Fishmonger's Swallet is a small stream sink located near the village of Alveston in the Vale of Berkeley, South Gloucestershire. Initial clearing of the swallet was undertaken by Clive Grace, a local fishmonger, from whom the site derives its name. Subsequently, David Hardwick and the Hades Caving Club assumed control of the dig, revealing a substantial quantity of disarticulated human and animal bones, particularly canids, concentrated within an area designated the Bone Idle Chamber (*59*). This discovery attracted the attention of the Channel 4 programme Time Team and a three-day archaeological investigation conducted by the team uncovered additional human and animal bone fragments, along with evidence of post holes on the surface near the swallet (*60*).

Post-excavation analysis of the human remains by Cox and Loe (*61*) showed a minimum number of five individuals, but possibly six adult females and five adult males represented by the assemblage. The degree of fragmentation and environmental staining is limiting for macroscopic observations, nevertheless some evidence of pathology (Paget's disease, degenerative joint disease and abscesses) and perimortem trauma was seen on some elements (*61*).

It is important to note that a small creek flows into the swallet from the surface, carrying modern debris down a short vertical shaft into the Bone Idle Chamber. The co-mingling of

modern and archaeological material means it is impossible to interpret the depositional history of the site stratigraphically, so radiocarbon dating is essential. A total of sixteen radiocarbon dates have been obtained from skeletal material and recent programme comprising seven human (BRAMS-5057, BRAMS-5058, BRAMS-5059, BRAMS-5060) and six canid (BRAMS-5061, BRAMS-5062, BRAMS-5063, BRAMS-6671, BRAMS-6672, BRAMS-6673) bone have produced dates centred on the later Iron Age (*62*).

Canterbury: Sample C11907 comprised dentine powder taken from a left maxillary third molar from a disarticulated complete cranium (CW29) held in the archives of Canterbury Archaeological Trust (CAT) in Canterbury, Kent. There was some uncertainty about the provenance of the cranium at the time it was sampled. The site code was associated with the excavations at Whitefriars Shopping Centre, Canterbury which uncovered two main phases of activity associated with the deposition of human remains: a Roman ditch and a cemetery associated with a late medieval Augustinian friary which was founded in 1324 CE and dissolved in 1538 CE (*22*). It was initially unclear which phase of deposition CW 29 belonged to. A sample of dentine from the same tooth analysed for DNA produced a radiocarbon determination of 716±25 BP. The $\delta^{13}C$ value from the Accelerator Mass Spectrometer (-18.6) indicated that a portion of this individual's dietary protein was derived from marine resources, which is likely to have affected the radiocarbon date via the marine reservoir effect. When the raw radiocarbon determination was calibrated using a mixed Marine20/IntCal20 curve with 28±10% marine protein and ΔR value of 0±50, this produced a range of 736-536 years ago (1258-1461 cal. CE), consistent with the dates of the Augustinian cemetery. The excavated graves primarily came from the Lay cemetery and therefore CW 29 is likely to have belonged to one of the general population rather than one of the Augustinian monks.

Poulton: Sk 435 was recovered during the excavation of a medieval burial ground and associated chapel in rural Poulton, Cheshire, England. The site was used for interment of individuals from a small farming community, with historical documents and radiocarbon dates indicating use between the 13th and 16th centuries CE (*24*, *63*). The remains are curated at Liverpool John Moores University as part of their teaching and research collection. Sk 435 was identified osteologically as a mature adult of undetermined sex, who was approximately 40-44 years old at the time of death. The body was laid out in typical medieval fashion, extended in a supine position with west–east orientation. Neither grave goods nor a burial marker were present. Continual re-use of the cemetery resulted in the individual's legs and portions of the pelvis being removed by later interments. Radiocarbon dating of the remains provided a date of 1290-1390 cal. CE at 2 δ (Wk52986. 646 ± 14BP).

Sampling, DNA extraction and library preparation

One tooth from each individual included in this study was sampled and processed in a dedicated cleanroom facility at the Francis Crick Institute. An EV410-230 EMAX Evolution Dentistry drill was used to clean the surface of the tooth and both the cementum and multiple fractions of the dentine were sampled, resulting in ~11- 35 mg of powder from the dentine. 300 µl (<10 mg of powder), 600 µl (10-25 mg) or 1,000 µl (>25 mg of powder) of extraction buffer (0.5 EDTA pH 8.0, 0.05% Tween-20, 0.25 mg/ml Proteinase K (*64*)) was added to the dentine powders and incubated for 24 hours at 37°C. They were then centrifuged for 2 minutes at 16,400g (13,200 rpm) in a table centrifuge and 140 µl of the supernatant was transferred into LVL tubes for automated extraction on an Agilent Bravo Workstation (*65*). Extracts were turned into single-stranded DNA libraries (*66*), then double-indexed (*67*) and underwent paired-end sequencing with a 2×100 paired-end read configuration on the Illumina HiSeq4000,

NextSeq500 and NovaSeq 6000 platforms (**Table 1** for sequencing effort per library). All samples were processed alongside negative extraction controls as well as positive and negative library controls.

One library, C10416 from Wetwang Slack, underwent size selection to remove fragments shorter than 35 bp and longer than 150 bp, as in Gansauge et al. 2020 (*66*). Specifically, 100 ng of the initial library was biotinylated and streptavidin beads were used to isolate the non-biotinylated strand and obtain a single-stranded library. This sample was then loaded on a denaturing polyacrylamide gel along with 35 bp and 150 bp insert markers, and fragments within the desired sequence length were physically excised and eluted from the gel, after overnight incubation. The resulting size-selected libraries were further amplified and sequenced on an Illumina NovaSeq 6000 instrument.

Bioinformatic Processing, Metagenomic Screening and Authentication

Samples were initially processed via the nf-core/eager v2 pipeline (*68*). First, adapters were removed, paired-end reads were merged and bases with a quality below 20 were trimmed using AdapterRemoval v2 (*69*) with –trimns –trimqualities –collapse –minadapteroverlap 1 and –preserve5p. Merged reads with a minimum length of 35 bp were mapped to the hs37d5 human reference genome with Burrows-Wheeler Aligner (BWA-0.7.17 aln) (*70*) using the following parameters "*-l 16500 -n 0.01*" (*19, 71*). We then analysed sequences that did not align successfully to the human genome using Kraken2 (*56*) and identified individuals as putatively positive for *B. recurrentis* by assessing an excess number of observed *k*-mers (sequence matches).

These libraries were subsequently aligned to the *B. recurrentis* A1 reference genome (chromosome and plasmids; GCF_000019705.1) using BWA-0.7.17 aln (*70*) parameters "*-l 16500 -n 0.01 -o 2*" and duplicates were removed using MarkDuplicates from the Picard toolkit. We assessed the authenticity of the final set of sequences using the following criteria (*27*): i) the observation of postmortem damage, ii) the number of sequences being negatively correlated with edit distance from the reference genome, and iii) an unimodal fragment length distribution via DamageProfiler (*72*) iv) even breadth of coverage across the *B. recurrentis* A1 reference genome using SAMTools v1.3.1 *depth (73)*. Additionally, these libraries were aligned to the *B. duttonii* Ly and the *B. crocidurae* DOU reference genome (NC_011229.1 and NZ_CP004267.1 respectively) and their edit distance distributions were compared (**Figure S2**). Screening libraries that passed these authentication criteria, were taken forward for further shotgun sequencing. For the final BAM files, we merged shotgun BAM files using SAMTools *merge* resulting in a final chromosome coverage of 29.4X, 11.2X, 3.5X and 0.8X coverage for C10976 Poulton, C10416 Wetwang Slack, C13361 Fishmonger's and C11907 Canterbury, respectively when aligned to *B. recurrentis* A1 (**Table 1, Figure S1**).

Dataset Curation

All published genomes used in this study are listed in **Table S1**. This includes all modern genome assemblies available from NCBI (accessed April 2024). In addition, we *de-novo* assembled six modern genomes available on the SRA linked to BioProject PRJNA378726 using UniCycler v0.50 in short-read only mode, recovering high quality assemblies (N50>700,00 in all cases). The previously sequenced ancient *B. recurrentis* genome OSL9 (*18*) was downloaded from ENA after which adapters were removed and reads were merged using

AdapterRemoval2 and processed identically to the described method for individuals in this study (**Methods, Bioinformatic Processing**).

Alignment Approaches and Phylogenetic Reconstruction

Two approaches were used to construct an alignment for phylogenetic inference. The first used a reference mapping approach to the *B. recurrentis* A1 reference genome (as described above). The second was to construct a core gene reference alignment, built using an assessment of gene content in modern strains. For the latter, we initially applied Panaroo v1.1.2 (*28*) on the 11 (seven *B. recurrentis*, two *B. duttonii* and two *B. crocidurae)* modern RF genomes and assemblies available specifying the *–core* flag in relaxed *-mode* to obtain a core alignment of genes featuring in 99% of considered genes. We then aligned all genomes (ancient and modern) to the core gene sequence using BWA-0.7.17 *aln* parameters "*-l 16500 -n 0.01 -o 2*", and processed to remove duplicates by keeping only the first sequence in case multiple sequences had the same start and end positions (https://github.com/pontussk/samremovedup). For both approaches, for published assemblies, where short-reads were not available, we applied SeqKit (*74*) to generate pseudo-reads for which the mapping pipeline could be applied with 45bp fragment size and a sliding window of 5bp.

In both cases, modern genomes were converted to fasta sequences keeping all bases with a coverage of 1 using HTSBOX (*75*). For the ancient genomes, we computed Base Alignment Qualities using SAMTools "*mpileup -E*", restricted to a minimum phred-scaled mapping quality of 30 and base quality 30 using SAMTools v1.3.1. The MD field was modified to record mismatches to the reference using SAMtools *calmd*. Relative to the A1 reference genome and core genome alignment, C to T transition mutations on the forward strand and G to A transition mutations on the reverse strand were masked to correct for the possible effects of cytosine deamination in the single-stranded ancient DNA sequences (https://github.com/pontussk/mpileup2consensus.py/blob/main/mpileup2consensusfasta.py, using the option --ssDNAlib_refcall). Additionally, using this tool we filtered out all heterozygous base calls (--minsupport 1.0) and only kept sites with a minimum coverage of 3 calls per site (--mindepth 3), resulting in a filtered fasta for each ancient genome.

Ancient and modern consensus sequence files in fasta format were then concatenated and polymorphic positions were identified for the initial maximum likelihood phylogeny. Using https://github.com/pontussk/fasta_nomissing.py, polymorphic positions with a threshold of maximum missingness of 20% per site and a maximum missingness of 20% per genome were retained (--maxmissing 0.2 --maxmissing_ind 0.2), resulting in 4,200 sites when aligned to the core-genome (**Figure S3**) and taken forward for maximum likelihood phylogenetic reconstruction in IQ-TREE v.1.6.12. Due to these thresholds, C11907 Canterbury was excluded from most downstream analyses. We implemented model testing using the ModelFinder in IQ-TREE (*76*), which suggested a TIM+F+ASC as the best-fit model according to the Bayesian Information Criterion. We implemented 1000 rapid bootstrap replicates and rooted the maximum likelihood phylogeny in FigTree (*77*) using *B. duttonii* Ly as an outgroup.

Recombination and Temporal analysis

Using the initial SNP tree and the whole core-genome alignment, ClonalFrameML v1.13 (*29*) was applied to detect homoplasies and putative recombination tracts. Identified recombinant tracts were masked from the alignment **(Figure S5)**.

The recombination pruned core genome alignments and corresponding phylogenies were inspected for signatures of temporal evaluation using the *roototip()* function implemented in BactDating (*30*), evaluating empirical significance following 10,000 randomisations of the sampling dates. Temporality was assessed for a dataset including and excluding the C13361 Fishmonger's sample due to its low coverage, and using phylogenies built on alignments with and without the inclusion of transitions. In all cases we obtained a highly significant temporal regression (**Table S2**).

Resulting alignments were subsequently taken forward for formal Bayesian tip-dating calibration implemented in the BEAST2 workflow (*31*). In all cases, variant positions were considered (with corresponding correction for the base composition of invariant sites) with the prior on the tip dates corresponding to the date of sample collection, or, where a range of dates was given (either in contemporary samples or corresponding to radiocarbon dates) the mean estimate was used as an initial prior. Following evaluation of possible substitution models in BModelTest (*78*), in all cases a GTR substitution model was best supported and selected for further analysis. BEAST2 was run assuming either of a strict or relaxed (exponential distribution) molecular clock exploring three distinct priors for the demographic model: coalescent constant, coalescent exponential and coalescent bayesian skyline, specifying 50 million chains sampling every 1000. Resulting chains were inspected for convergence in TRACER, requiring an effective sampling space (ESS) of >200, with resulting posterior estimates extracted following discarding the first 10% of chains as burn-in. All runs exhibited a significant difference between the posteriors obtained when sampling from the prior (data absent model). Finally, model fit was assessed using the path-sampling model, requiring 100 steps over 250,000 chains, to establish marginal likelihoods for the models and corresponding Bayes Factors. All results are provided in **Table S3** with posterior distributions available in **Figures S7** and **Figures S8**.

We also took an alternative approach to approximating the divergence time, which excludes external branches private to ancient genomes, and thus removes any potential impact of sequence errors unique to the ancient genomes. To identify ancestral and derived variants separating modern *B. recurrentis* from the common ancestor with *B. duttonii* Ly (**Table S4**), we used the core-genome aligned multifasta file containing the ancient genomes which had been filtered using mpileup2consensusfasta.py and all modern *B. recurrentis*, *B. duttonii* Ly and *B. crocidurae* DOU genomes and generated a VCF using 'SNP-sites' (*79*). We then filtered the VCF to keep only SNPs where all modern *B. recurrentis* genomes matched the *B. recurrentis* A1 genome, and ancestral where both *B. duttonii* and *B. crocidurae* were identical to each other but differed from the *B. recurrentis* variant. We calculated the number of variants that were missing, ancestral or derived in our ancient genomes (**Table S4**) and used the proportion derived and the approximate date of the ancient genomes to identify an approximate TMRCA based on the intercept of linear regression.

Pangenome Analysis, Ancestral State Reconstruction and Evaluation of Gene Content

We constructed a full pan-genome given the diversity in modern *B. recurrentis*, *B. duttonii* and *B. crocidurae* genomes (**Table S1**). To do so we initially applied Prokka v1.12 (*80*) to generate gene annotations for each modern genome. Panaroo v1.1.2 with the *-pan* flag in relaxed mode

and otherwise default thresholds was implemented and then used to identify gene clusters and create a list of genes present in all of the given modern genomes (*28*). We identified 14,475 genes and gene clusters across all modern relapsing fever *Borrelia* species (**Table S9, Figure S13)**. Among the modern *B. recurrentis* genomes, Panaroo identified ~1,100 of these genes as present. We additionally conducted a smaller-scale pan-genome reconstruction including only members of *B. crocidurae, B. duttonii* and *B. recurrentis* following the same steps, estimating a total of 3,184 genes.

To assess properties of genome gain and loss by species we considered the unique and intersecting gene count from the relapsing fever-wide pan-genome matrix (**Table S9**) for each of *B. crocidurae*, *B. duttonii* and *B. recurrentis* using the two genome assemblies available in the former two cases and the *B. recurrentis* A1 reference genome paired with a randomly selected assembly in the latter (SRR5332755). In addition, to estimate the ancestral pan-genome complement of each species, we applied discrete trait ancestral character estimation implemented in the *ace()* function in the R package Ape v5.7-1 (*81*). To do so we considered the 2,265 variable genes in the 3,184 gene pan-genome and the phylogeny computed over the core gene alignment, selecting a maximum likelihood implementation to generate a probability of each gene being observed at the relevant ancestral node in the tree. Genes with an inferred probability of 0.5 were discarded from consideration resulting in probabilistic assignments of 2,165 genes across the three species (**Table S6**).

To identify the presence and absence of genes in the pan-genome we aligned simulated short-read modern FASTQs (see SeqKit simulation parameters above) using BWA-0.7.17 *mem* with the parameters "*-B 40 -O 60 -E 10 -L100*" to the pangenome and the ancient FASTQs using BWA *aln* with the parameters "*-l 16500 -n 0.01 -o 2*". We then used the pangenome gene clusters. This allowed us to set an arbitrary threshold to identify genes as present required a minimum threshold of 70% coverage and below 30% coverage for absence. In addition we only kept genes which showed phylogenetically congruent patterning, resulting in consideration of 71 well-resolved genes of interest **(Table S7**). We performed *in silico* functional predictions for these genes using the EggNOGG-Mapper web server (http://eggnog-mapper.embl.de/) and InterproScan (*82*), both with default parameters. Putative gene functions were inferred from the annotations provided by both tools **(Table S10).** The gene neighbourhood of identified hits, including *Soj_1* and *ParA*, were assessed using the Panaroo pan-genome network graphs, visualised in Cytoscape v3.10.2. In this case, gene homology was obtained by translating the nucleotide sequence of *Soj_1* and *ParA* to amino acids before aligning in MUSCLE (*83*) and calculating pairwise identity.

Visual inspection of SNPs and Indels

An additional reference-based analysis was also carried out, aligning all genomes to *B. recurrentis* A1 and *B. duttonii* Ly. Here, all ancient and simulated short-read modern genomes were aligned to each of the aforementioned references using the described BWA aln pipeline for ancient samples and BWA *mem* for the modern genomes (specifying the same parameters as when aligning to the pangenome). After the removal of duplicates, we assessed the coverage across both chromosomes, plasmids, and previously reported genes, and manually inspected previously reported SNP mutations using Integrated Genome Viewer (IGV) (**Figure S11**). Additionally, we assessed regions of missingness by identifying regions with a gap in coverage larger than 500bp in the ancient genomes using BEDTools (*84*) when aligned to each of the *B. recurrentis* A1 reference genome and the *B. duttonii* Ly reference genome (**Figure S14**, **Table S11**). We noted some marked drops in coverage consistent with genomic deletions or

rearrangements. As an example, despite the low coverage of C11907 Canterbury, we could confirm a unique 26.1kb deletion when aligned to both colinear plasmid pl124 in *B. recurrentis* A1 and pl165 in *B. duttonii* Ly (**Figure 1B, Figure S15, Table S11**). The pl124 plasmid in *B. recurrentis* A1 has been previously observed to exhibit a ~40kb deletion in comparison to the *B. duttonii* plasmid (*3*). Marosevic *et al*. (*8*) identified this 40 kb region to also be present in eight modern-day *B. recurrentis* genomes from East Africa, suggesting that this deletion may be a recent event, or potentially the product of an incomplete assembly of the A1 reference genome plasmid. We find this 40kb region is also present in all of our ancient *B. recurrentis* observations, consistent with these proposed scenarios.

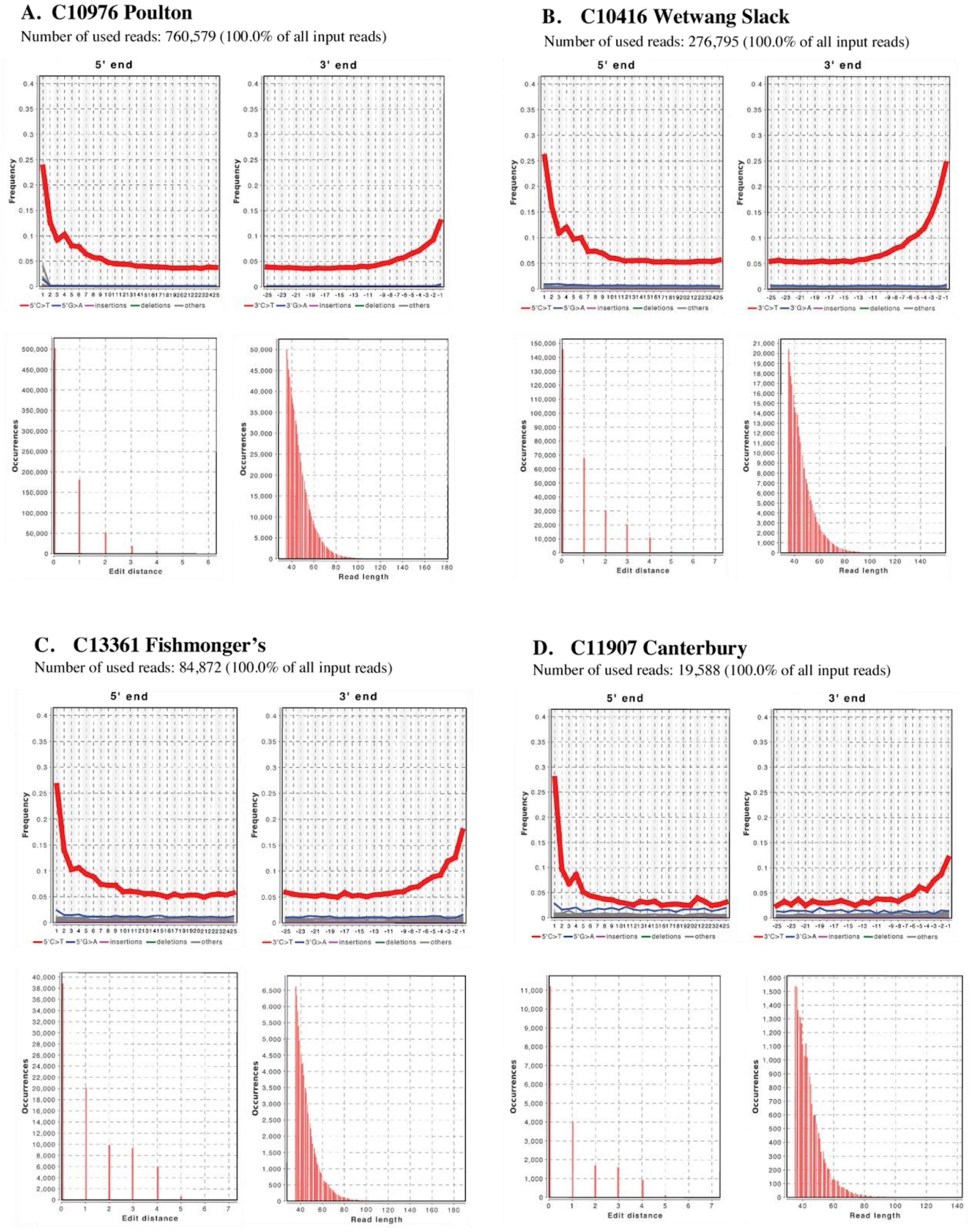

Figure S1. Ancient pathogen authentication of four ancient genomes aligned to the *B. recurrentis* A1 reference. Edit distance, damage and fragment length distribution for the concatenated genomes for each sample when aligned to *B. recurrentis* chromosome and plasmids (A1 reference genome) via DamageProfiler (*72*). **a)** C10796 from Poulton **b)** C10416 from Wetwang Slack **c)** C13361 from Fishmonger's **d)** C11907 from Canterbury.

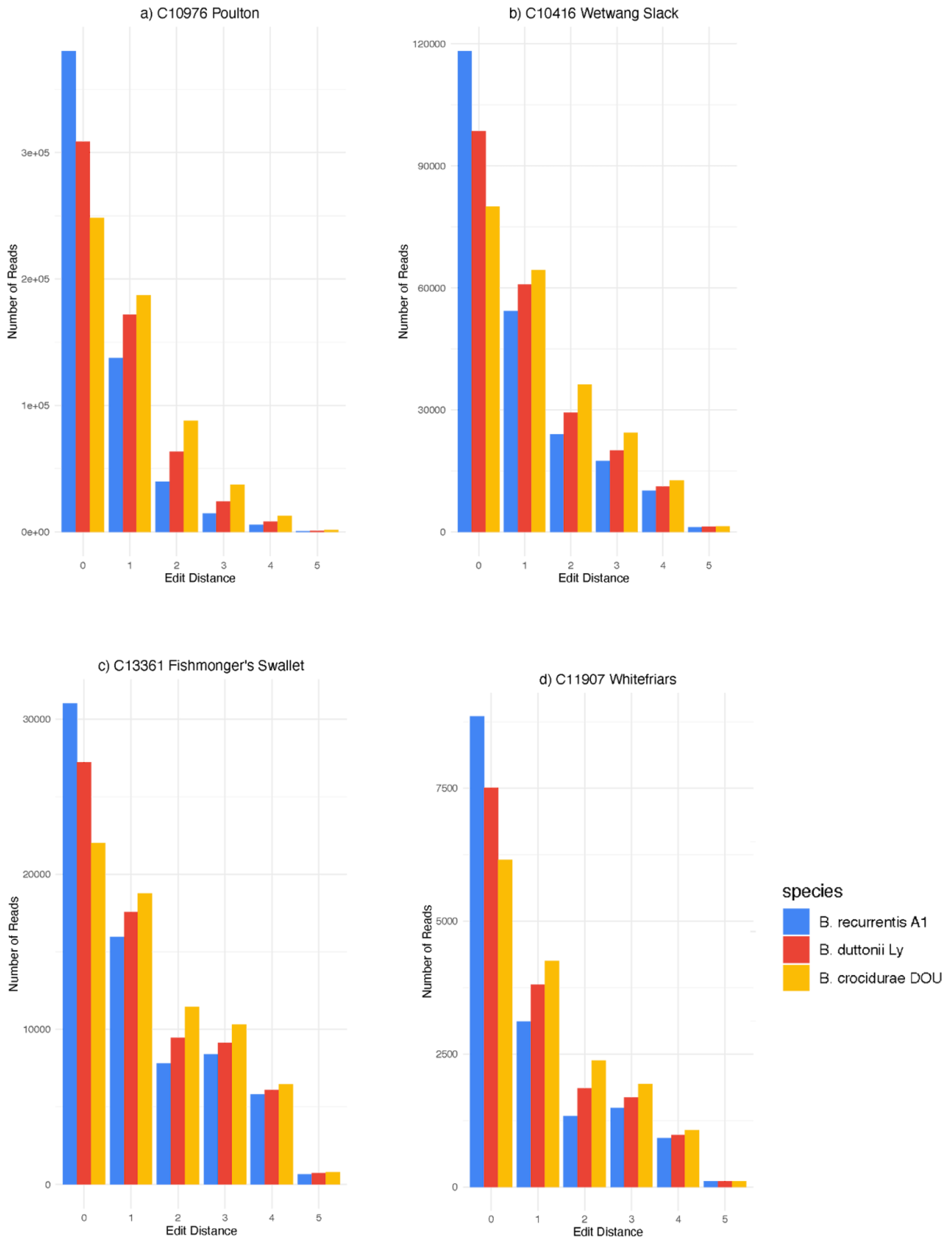

Figure S2. Edit distance distribution of four ancient genomes from this study when aligned to *B. recurrentis* A1, *B. duttonii* Ly, and *B. crocidurae* DOU reference. Edit distance (x-axis) against number of reads (y-axis) of all newly considered ancient genomes from this study, when aligned to *B. recurrentis* A1 (blue), *B. duttonii* Ly (red) and *B. crocidurae* DOU (yellow) reference genomes. **a)** C10796 from Poulton **b)** C10416 from Wetwang Slack **c)** C13361 from Fishmonger's **d)** C11907 from Canterbury

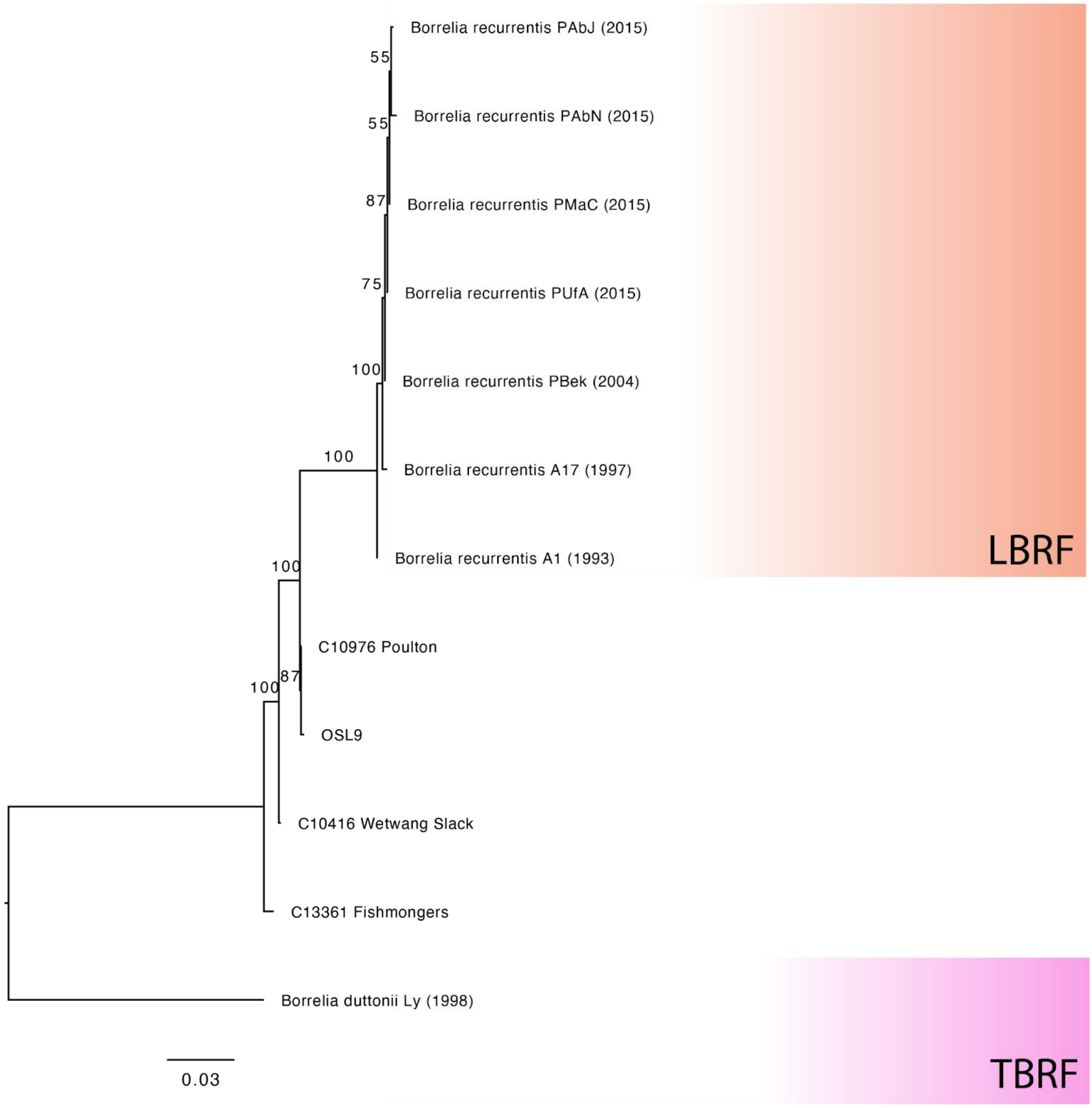

Figure S3. Maximum Likelihood SNP phylogeny of ancient and modern *B. recurrentis* genomes and *B. duttonii* outgroup when aligned to the Panaroo core-genome. Maximum likelihood phylogenetic tree (GTR+F+ASC according to AIC) constructed on variability over the core genome SNP alignment of 2,006 sites filtering for 20% missing per-site and per-genome including transitions.

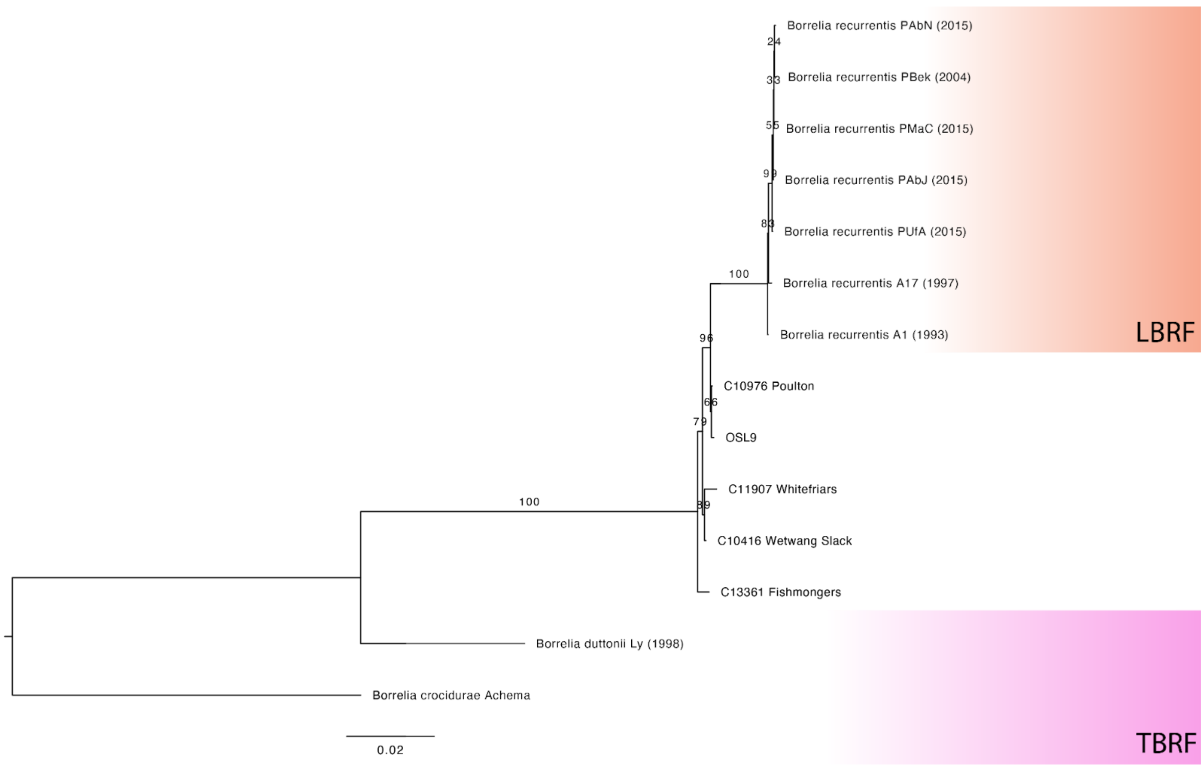

Figure S4. Maximum Likelihood SNP phylogeny of ancient and modern *B. recurrentis* genomes and *B. duttonii Ly* outgroup when aligned to *B. recurrentis* A1 reference genome with relaxed filtering thresholds. Maximum likelihood phylogenetic tree (K3Pu+F+ASC model implemented with rapid bootstrapping) constructed on variability over a reference based alignment to the *B. recurrentis* A1 reference genome (3,354 sites, 20% missingness per site, maximum missingess across genome 90%). All ancient genomes were filtered for a minimum per-site fold coverage of 3 except for C11907 Canterbury which was filtered for a minimum coverage of 2.

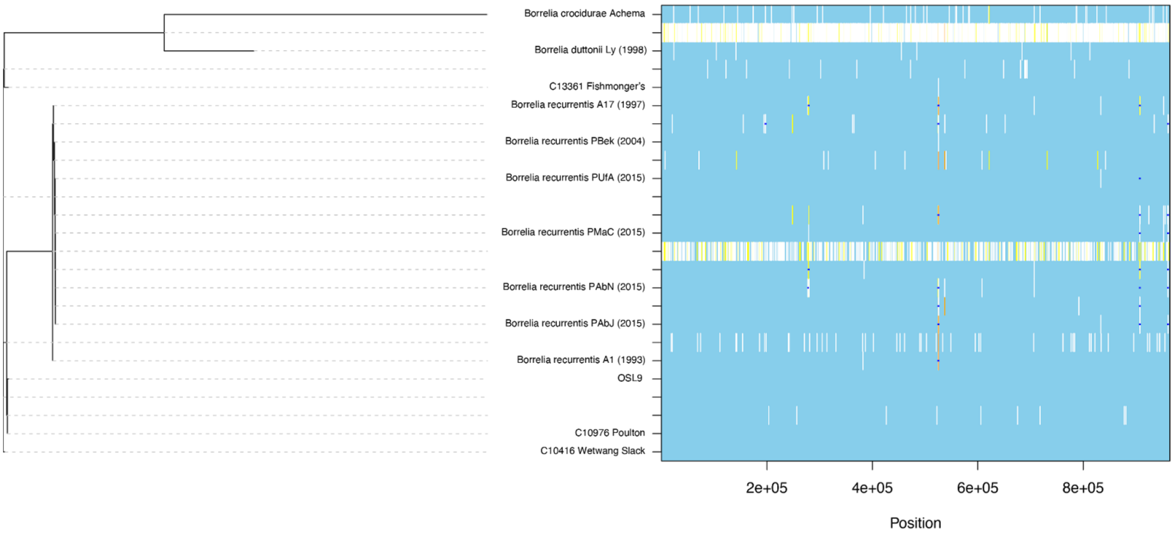

Figure S5. ClonalFrameML homoplasy recombination analysis of core genome. Reconstructed substitutions (white vertical bars) are shown for each branch of the maximum

likelihood tree. Dark blue horizontal bars indicate recombination events detected by the analysis with yellow indicating homoplasic positions.

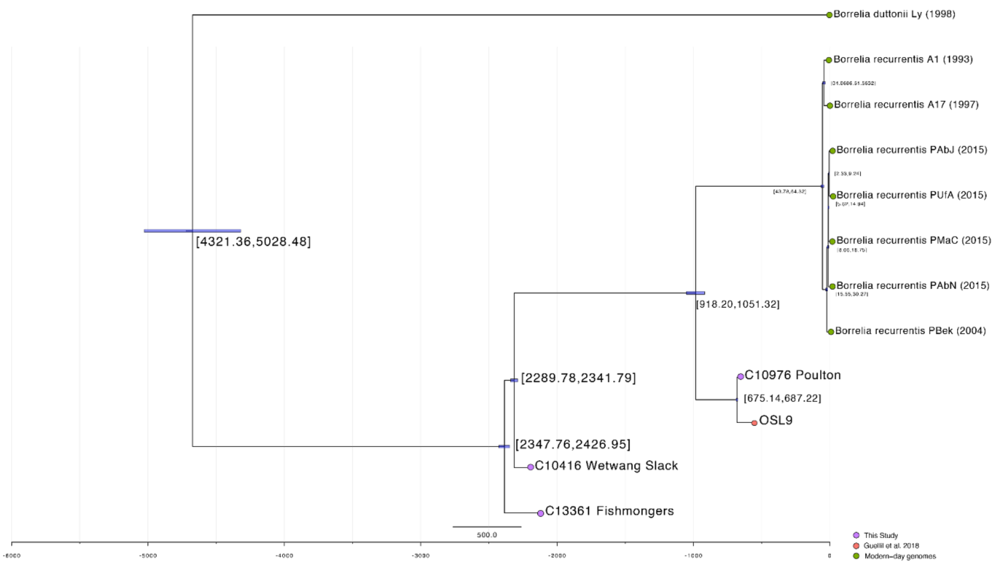

Figure S6. Beast2 tip-calibrated time tree when the C13361 Fishmonger's genome is included. Bayesian tip-calibrated maximum clade credibility time tree from Beast2, providing the best-supported model following path-sampling when C13361 Fishmonger's is included in the alignment. Confidence intervals around nodes provided the 95% higher posterior density. Ancient samples are highlighted by coloured tips.

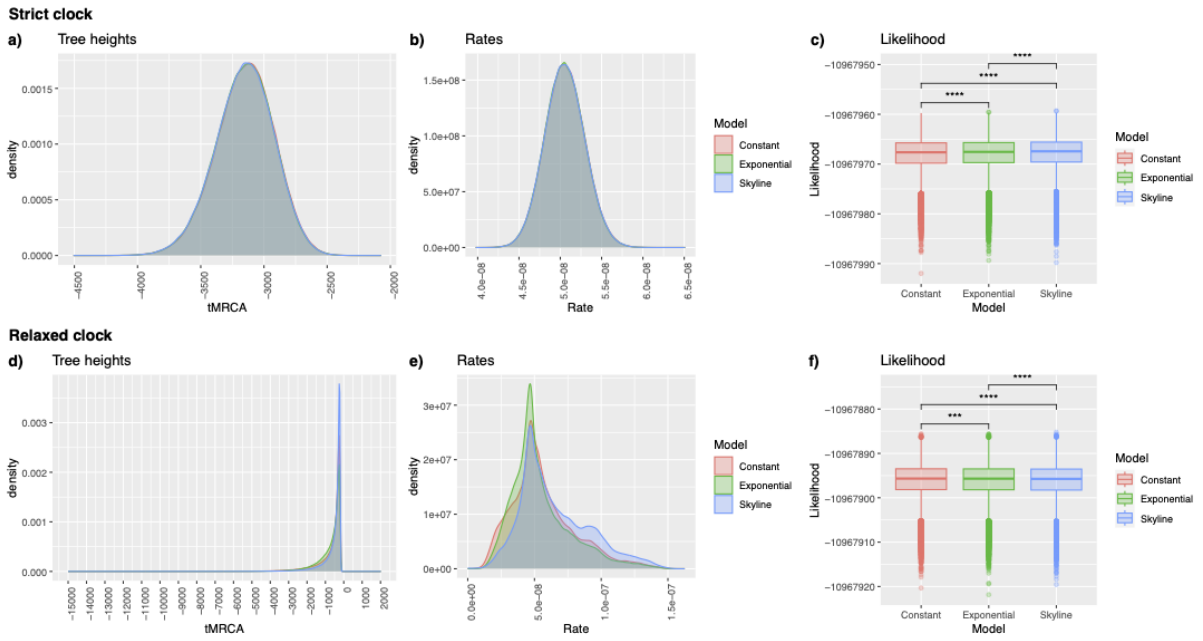

Figure S7. Posterior Distributions from Bayesian tip-dating analysis. Posterior distributions estimated under a strict clock (top row) and relaxed clock (bottom row) allowing three possible specifications of demographic models (red - coalescent constant, green - coalescent exponential, blue - coalescent skyline). Estimated tree heights are given in the left-hand panels (a,d), estimated clock rates are provided in the right-hand panels (b,e). Boxplots provide the posterior distribution of likelihood values under a strict (c) and relaxed (f) clock model.

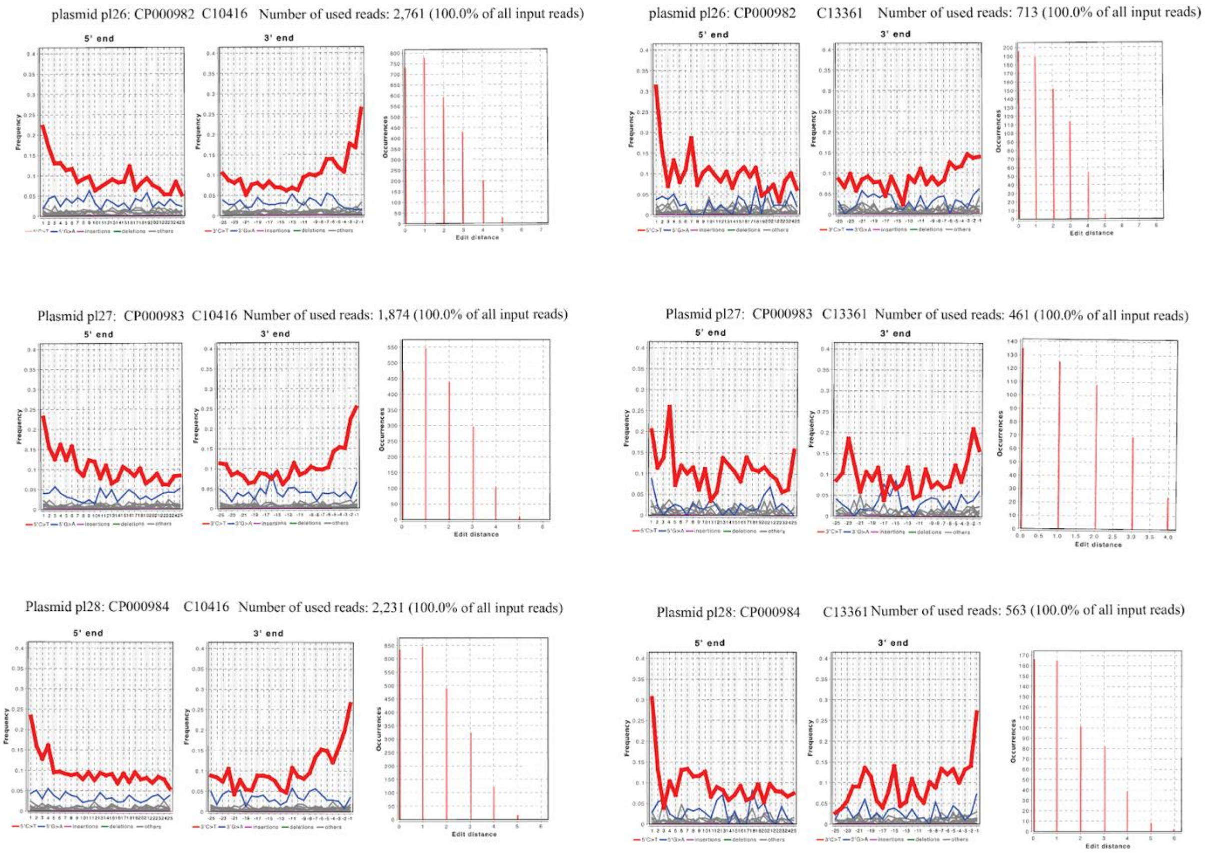

Figure S8. Plasmid authentication of *B. duttonii* plasmids pl26, pl27, pl28 in C10416 Wetwang Slack and C13361 Fishmonger's. Damage (deamination) pattern and edit distance of C10416 Wetwang Slack and C13361 Fishmonger's when aligned to *B. duttonii* plasmids pl26, pl27 and pl28 which are shown to be higher coverage than all other *B. recurrentis* genomes.

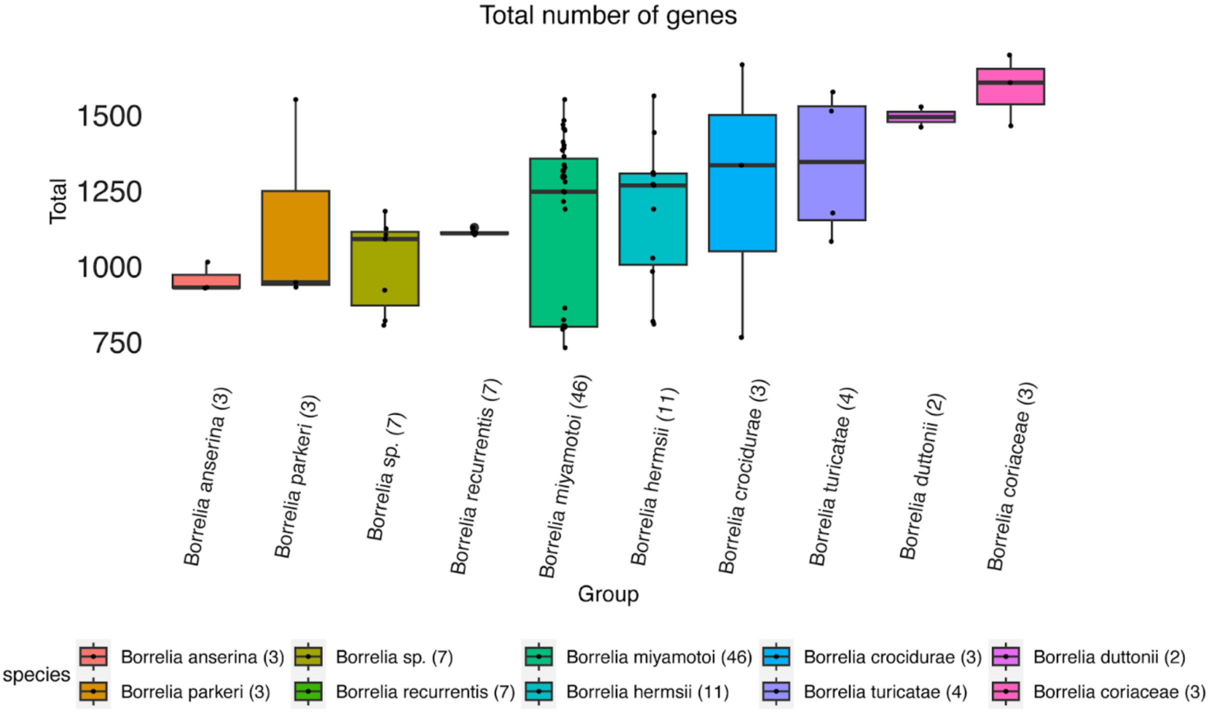

Figure S9. Per species distribution of the total number of genes identified across the *Borrelia-wide* pan-genome after filtering for truncated genes. The count of the number of genomes considered per species is provided in parentheses. Truncated genes were filtered using the Panaroo –*filter* parameter. Species where only one isolate was available was excluded from this plot.

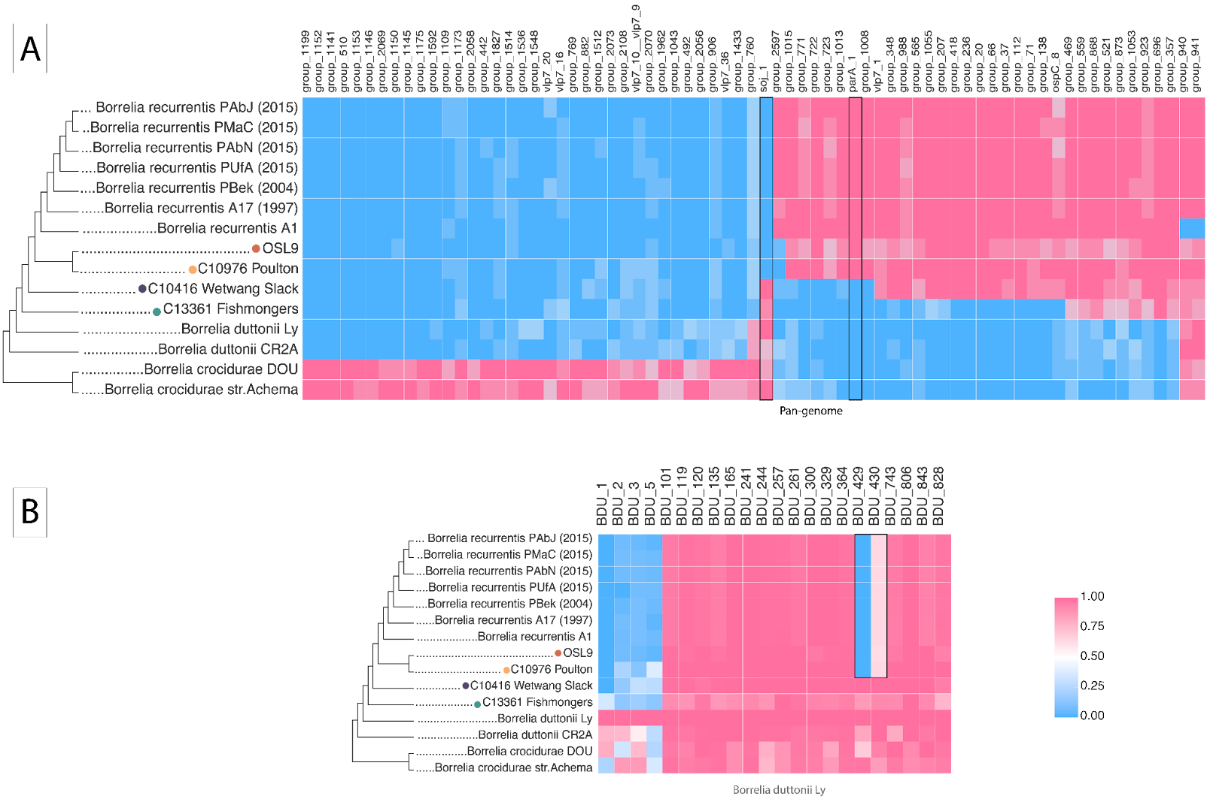

Figure S10. Presence/absence across pan-genome genes and gene clusters. A) Ancient and modern genomes were aligned to the pan-genome reference built on all modern *B. recurrentis*, *B. duttonii* and *B. Crocidurae* genomes (**Methods**). Normalised breadth of coverage across the gene and gene clusters was calculated using BEDtools v2.29.2. Genes that had a coverage between 0.3 and 0.7 were filtered out and genes that had a consensus coverage across the clade (modern *Borrelia*, medieval *Borrelia*) were kept. Additionally, genes that are in the same state (either all absent or all present) in the *B. duttonii* Ly and ancient and modern *B. recurrentis* were also filtered out. This resulted in 71 genes out of the 3,035 genes identified in the pan-genome output. Regions of interest highlighted in text are outlined with a black box. The cladogram provides the relationship between different genomes based on a SNP phylogeny. We provide gene-by-gene coverage in **Table S7. B)** We assess the normalised breadth of coverage of previously identified virulence genes in *B. duttonii* Ly (*3*) across ancient and modern *B. recurrentis* genomes.

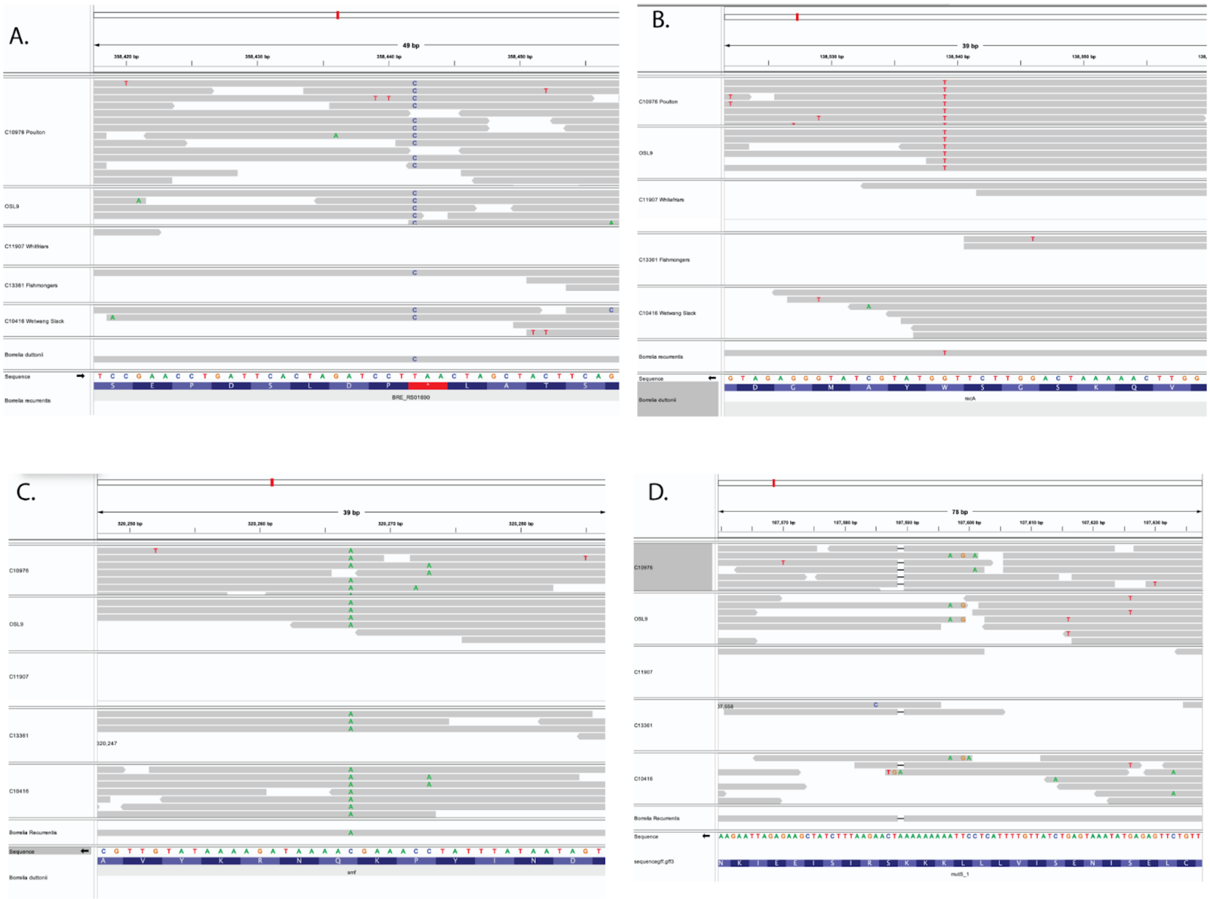

Figure S11. Integrated Genome Viewer of SNPs and INDELs. Previously identified SNPs and indels shown to be in different states in *B. recurrentis* A1 and *B. duttonii* Ly, visualised with IGV 2.17.4. A) Ancestral state of a polymorphism in the *oppA-1* gene in ancient samples from this study when aligned to *B. recurrentis* A1. The derived allele, which is present in *B. recurrentis*, results in an inframe stop codon. B) In-frame stop codon in the *recA* gene in *B. recurrentis* and medieval genomes, from a Tryptophan TGG (W) in *B. duttonii Ly* to a TAG stop codon (seen as a T on the forward strand), in the *B. recurrentis* A1 genome and the two medieval genomes included in this study. C10416 Wetwang Slack confidently shows the functional form of the gene, as does C11907 Canterbury but with the support of only 1 read. C) In-frame stop codon in the *smf* gene. IGV plot showing A on the forward strand in *B. recurrentis* A1 and all ancient genomes, resulting in a TAA in the reverse strand leading to an in-frame stop codon. The *B. duttonii* sequence is shown in the direction of protein synthesis. D) A frameshift mutation in the *mutS* gene, which is present in *B. recurrentis*, is represented by a deletion. Here we show the presence of reads aligning to the *mutS* gene with the same frameshift mutation present in *B. recurrentis A1*, also present in C10416 Wetwang Slack, C13361 Fishmonger's, and C10976 Poulton. Due to the low number of reads aligning to this region and the potential misalignment of the reads, it is unclear as to whether the frameshift mutation is absent or present in the other ancient individuals, or whether their presence is an artefact caused by misalignment.

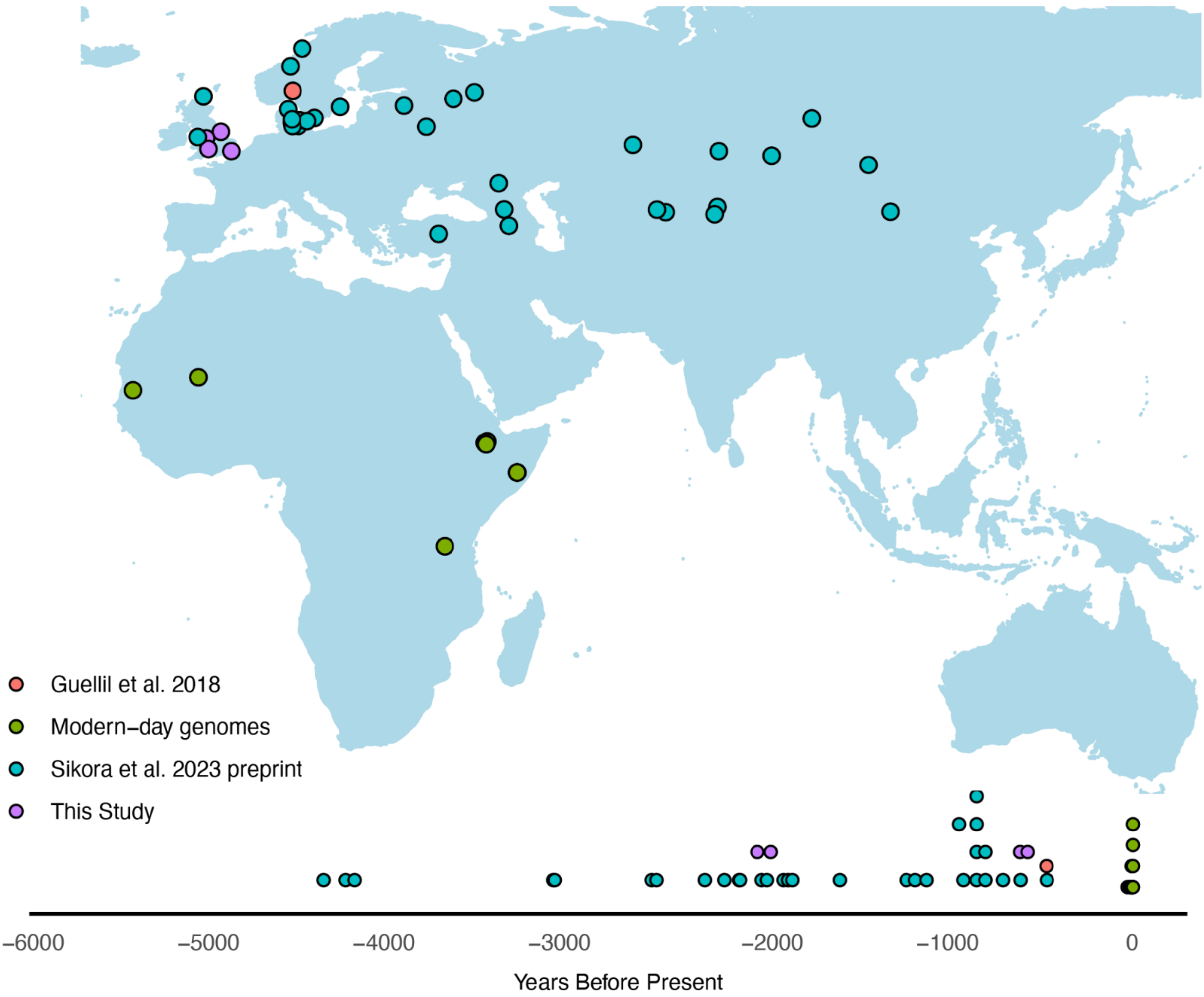

Figure S12. Map and timeline showing the distribution of ancient and modern *B. recurrentis* genome through space and time. Map providing the geographic location and timeline of *B. recurrentis* observations to date. Note that observations from the Sikora et al. 2023 preprint comprise hits assessed from partial recovery of sequencing reads as opposed to whole genome observations.

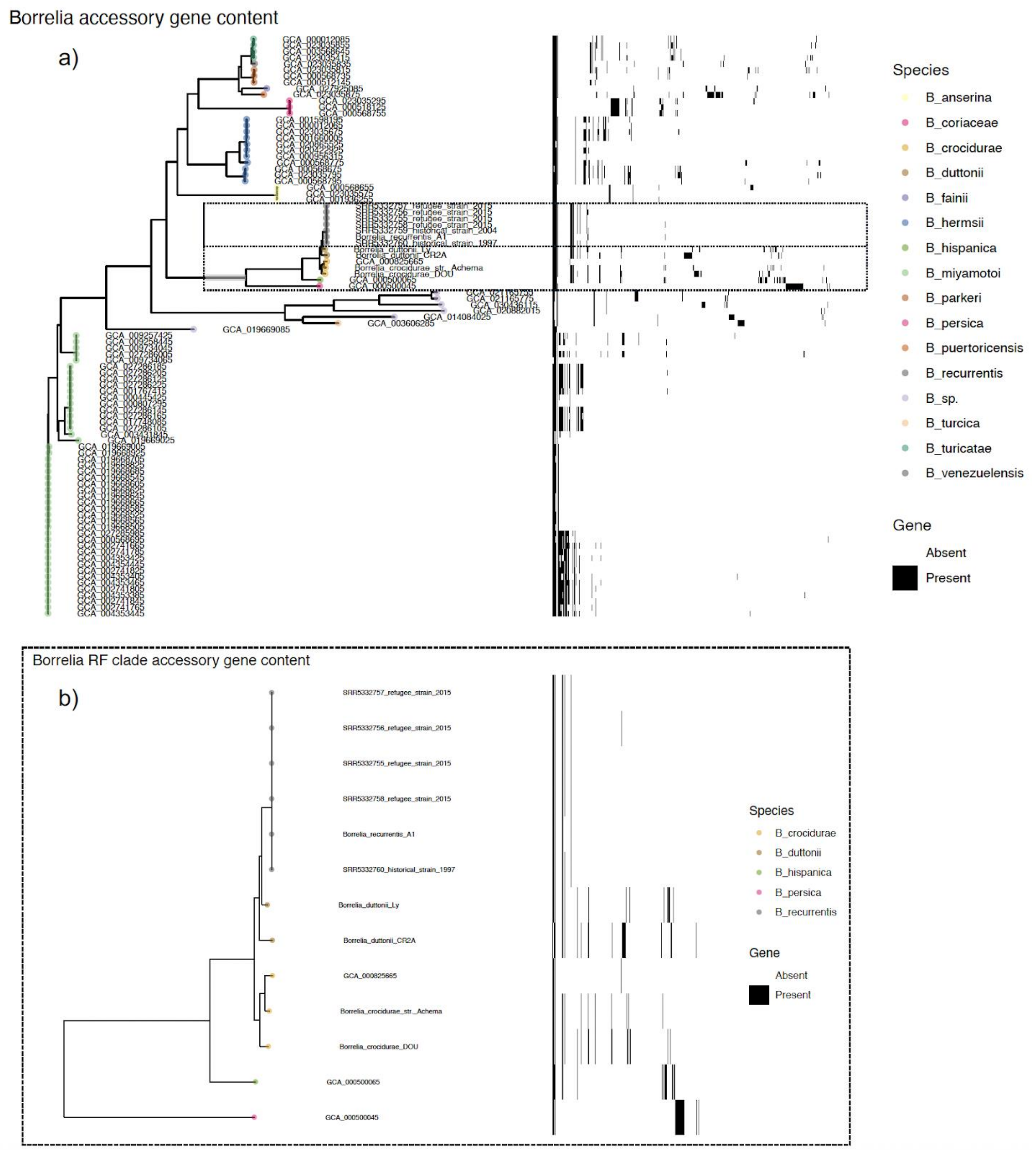

Figure S13. Pan-genome diversity across the *Borrelia* (RF) genus, comprising 14,475 genes. The phylogeny (right) provides a neighbour-joining tree representation based on the gene presence and absence over the 14,475 pan-genome **(Table S9).** The heatmap (right) provides the presence (black) and absence (white) across the pan-genome, with each column providing an individual gene.

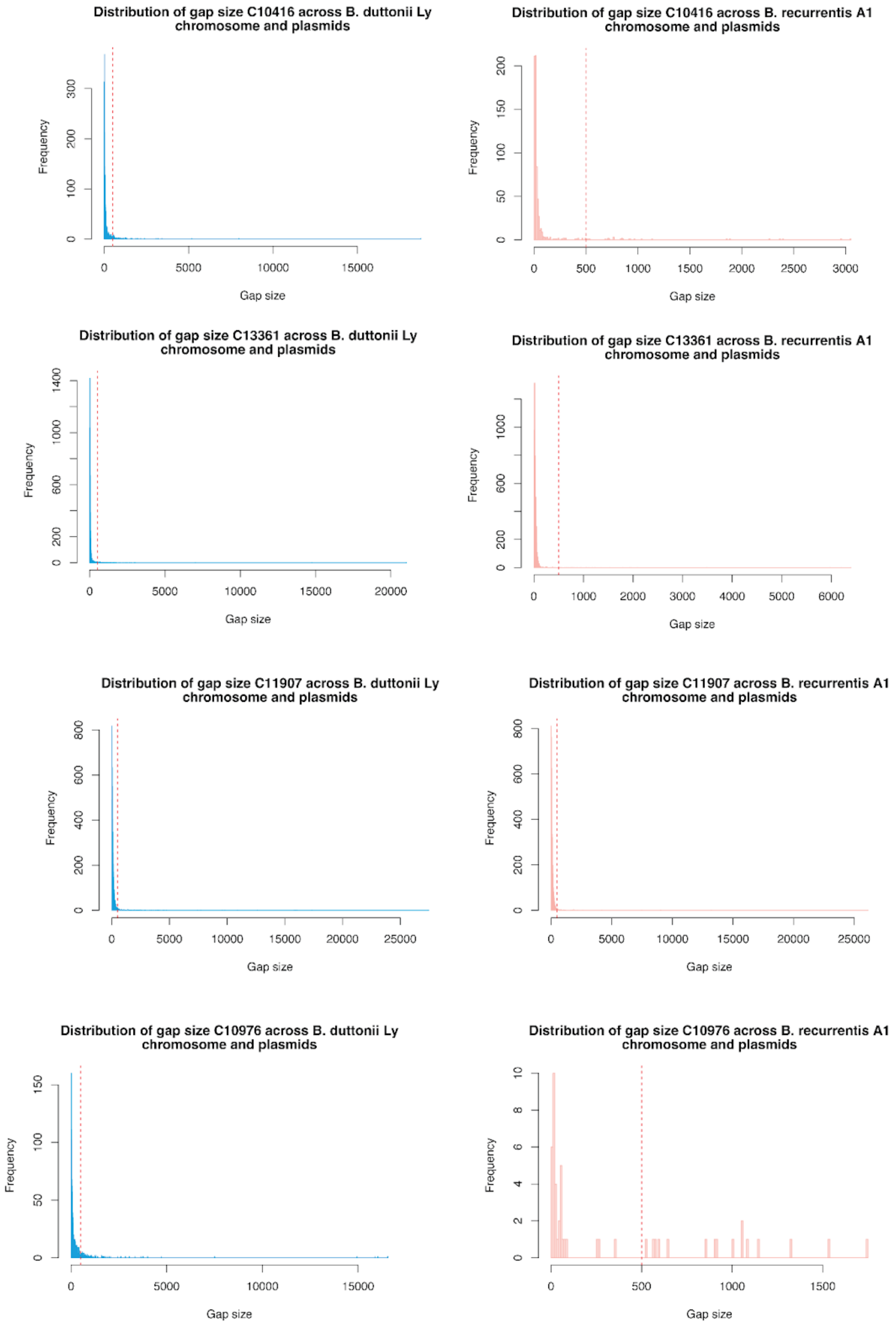

Figure S14. Gap size distribution plot to identify deletions over 500bp. Missingness distribution across ancient individuals from this study when aligned to the *B. duttonii* Ly

chromosome (blue) and *B. recurrentis* A1 chromosome and plasmids (pink). Positions of missingness window above 500bp (red dashed line) are reported in **Table S11**.

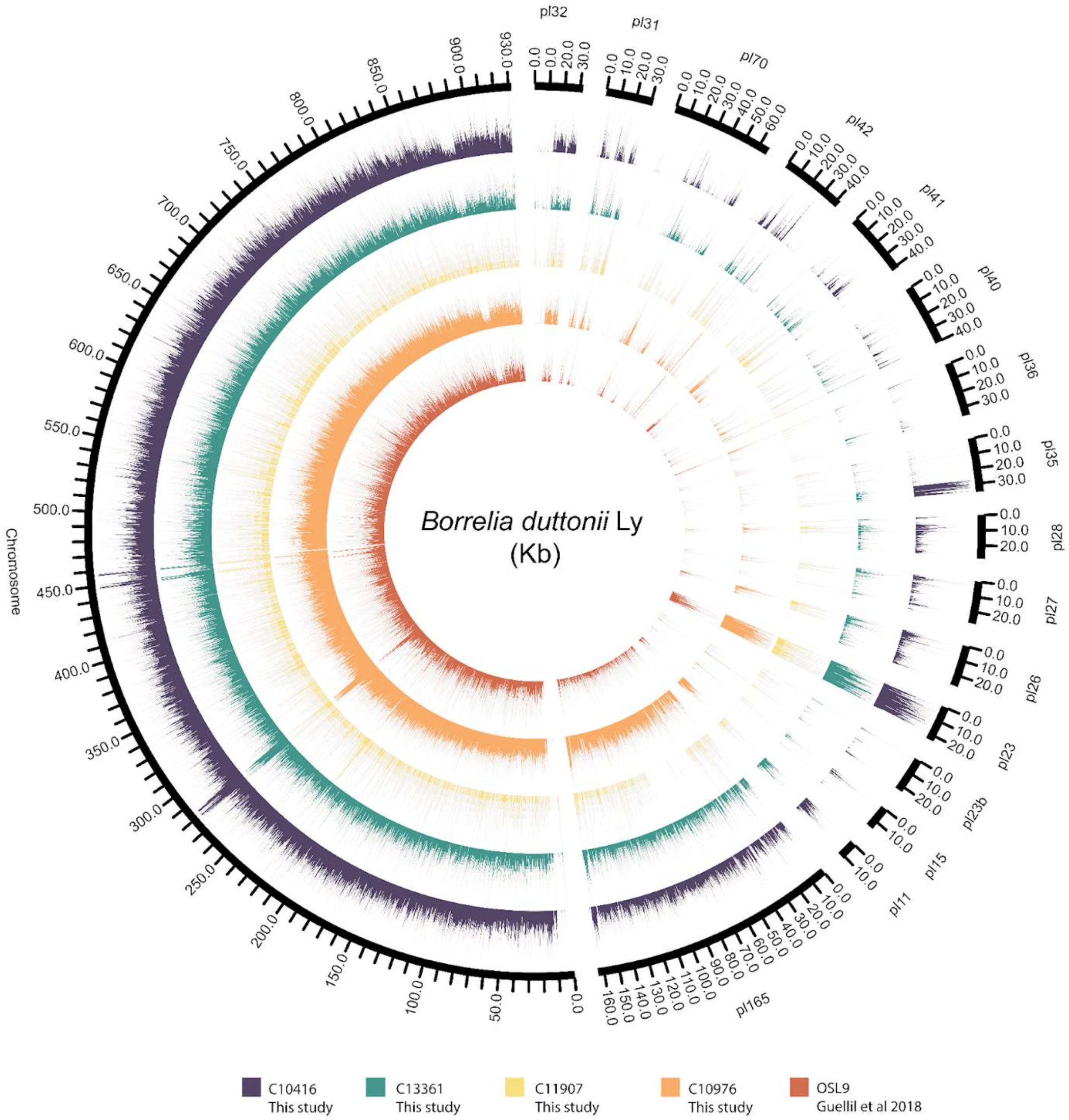

Figure S15. Circos plots of the per-sample coverage across the *B. duttonii* Ly reference genome. Circos plot of the coverage of ancient genomes across the *B. duttonii* chromosome and plasmids when aligned to the *B. duttonii* Ly reference genome (GCF_000019685.1). A window size of 100bp for the chromosome and 10bp for the plasmids was used to provide the normalised coverage per window plotted.

List of Supplementary Tables in Excel file

Table S1	All modern and ancient genomes used in this analysis with associated metadata.

Table S2	Assessment of temporal signal following the BactDating root-to-tip evaluation procedure.
Table S3	BEAST2 posterior probability estimates following assessment of the results over three demographic models tested using both strict and relaxed clock priors.
Table S4	Number of SNPs identified as ancestral versus derived in the ancient genomes.
Table S5	Coverage across plasmids when aligned to *Borrelia duttonii* Ly reference genome.
Table S6	Ancestral trait inferred probability of accessory gene carriage at each of the ancestral nodes of the core genome phylogeny.
Table S7	Normalised coverage after filtering for the 71 genes post filtering showing temporal patterning across our dataset.
Table S8	Normalised coverage of vmp carriage across all species when aligned to *Borrelia recurrentis* A1, *Borrelia duttonii* Ly, and *Borrelia crocidurae* DOU strains.
Table S9	Pan-genome across all relapsing fever species resulting in 14,475 identified gene clusters.
Table S10	Identified gene ontology annotations in the 71 filtered genes and gene clusters from EggNoGG and interproScan.
Table S11	Identified regions of missingness greater than 500bp when aligned to the *Borrelia recurrentis* A1 and *Borrelia duttonii* Ly chromosome.

References and Notes

1. J. O. Andersson, S. G. Andersson, Genome degradation is an ongoing process in Rickettsia. *Mol. Biol. Evol.* **16**, 1178–1191 (1999). doi:10.1093/oxfordjournals.molbev.a026208 Medline

2. C. M. Alsmark, A. C. Frank, E. O. Karlberg, B.-A. Legault, D. H. Ardell, B. Canbäck, A.-S. Eriksson, A. K. Näslund, S. A. Handley, M. Huvet, B. La Scola, M. Holmberg, S. G. E. Andersson, The louse-borne human pathogen *Bartonella quintana* is a genomic derivative of the zoonotic agent *Bartonella henselae*. *Proc. Natl. Acad. Sci. U.S.A.* **101**, 9716–9721 (2004). doi:10.1073/pnas.0305659101 Medline

3. M. Lescot, S. Audic, C. Robert, T. T. Nguyen, G. Blanc, S. J. Cutler, P. Wincker, A. Couloux, J.-M. Claverie, D. Raoult, M. Drancourt, The genome of *Borrelia recurrentis*, the agent of deadly louse-borne relapsing fever, is a degraded subset of tick-borne *Borrelia duttonii*. *PLOS Genet.* **4**, e1000185 (2008). doi:10.1371/journal.pgen.1000185 Medline

4. E. Talagrand-Reboul, P. H. Boyer, S. Bergström, L. Vial, N. Boulanger, Relapsing fevers: Neglected tick-borne diseases. *Front. Cell. Infect. Microbiol.* **8**, 98 (2018). doi:10.3389/fcimb.2018.00098 Medline

5. European Centre for Disease Prevention and Control (ECDC), Disease vectors (2017); https://www.ecdc.europa.eu/en/louse-borne-relapsing-fever/facts.

6. D. Raoult, V. Roux, The body louse as a vector of reemerging human diseases. *Clin. Infect. Dis.* **29**, 888–911 (1999). doi:10.1086/520454 Medline

7. L. Houhamdi, D. Raoult, Excretion of living *Borrelia recurrentis* in feces of infected human body lice. *J. Infect. Dis.* **191**, 1898–1906 (2005). doi:10.1086/429920 Medline

8. D. Marosevic, G. Margos, R. Wallich, A. Wieser, A. Sing, V. Fingerle, First insights in the variability of *Borrelia recurrentis* genomes. *PLOS Negl. Trop. Dis.* **11**, e0005865 (2017). doi:10.1371/journal.pntd.0005865 Medline

9. J. Rutty, *A Chronological History of the Weather and Seasons, and of the Prevailing Diseases in Dublin* (Robinson and Roberts, 1770).

10. G. Trevisan, M. Cinco, S. Trevisini, N. di Meo, M. Ruscio, P. Forgione, S. Bonin, *Borreliae* part 2: *Borrelia* relapsing fever group and unclassified *Borrelia*. *Biology* **10**, 1117 (2021). doi:10.3390/biology10111117 Medline

11. M. B. Shaw, A short history of the sweating sickness. *Ann. Med. Hist.* **5**, 246–274 (1933). Medline

12. P. Heyman, L. Simons, C. Cochez, Were the English sweating sickness and the Picardy sweat caused by hantaviruses? *Viruses* **6**, 151–171 (2014). doi:10.3390/v6010151 Medline

13. D. A. Warrell, Louse-borne relapsing fever (*Borrelia recurrentis* infection). *Epidemiol. Infect.* **147**, e106 (2019). doi:10.1017/S0950268819000116 Medline

14. M. Yimer, B. Abera, W. Mulu, B. Bezabih, J. Mohammed, Prevalence and risk factors of louse-borne relapsing fever in high risk populations in Bahir Dar city Northwest, Ethiopia. *BMC Res. Notes* **7**, 615 (2014). doi:10.1186/1756-0500-7-615 Medline

15. S. J. Cutler, Possibilities for relapsing fever reemergence. *Emerg. Infect. Dis.* **12**, 369–374 (2006). doi:10.3201/eid1203.050899 Medline

16. C. S. Pavia, "Immunologic detection of Lyme disease and the related borrelioses" in *Methods in Microbiology*, vol. 47, C. S. Pavia, V. Gurtler, Eds. (Academic Press, 2020), pp. 41–74.

17. S. J. Cutler, Relapsing fever Borreliae: A global review. *Clin. Lab. Med.* **35**, 847–865 (2015). doi:10.1016/j.cll.2015.07.001 Medline

18. M. Guellil, O. Kersten, A. Namouchi, E. L. Bauer, M. Derrick, A. Ø. Jensen, N. C. Stenseth, B. Bramanti, Genomic blueprint of a relapsing fever pathogen in 15th century Scandinavia. *Proc. Natl. Acad. Sci. U.S.A.* **115**, 10422–10427 (2018). doi:10.1073/pnas.1807266115 Medline

19. M. Meyer, M. Kircher, M.-T. Gansauge, H. Li, F. Racimo, S. Mallick, J. G. Schraiber, F. Jay, K. Prüfer, C. de Filippo, P. H. Sudmant, C. Alkan, Q. Fu, R. Do, N. Rohland, A. Tandon, M. Siebauer, R. E. Green, K. Bryc, A. W. Briggs, U. Stenzel, J. Dabney, J. Shendure, J. Kitzman, M. F. Hammer, M. V. Shunkov, A. P. Derevianko, N. Patterson, A. M. Andrés, E. E. Eichler, M. Slatkin, D. Reich, J. Kelso, S. Pääbo, A high-coverage genome sequence from an archaic Denisovan individual. *Science* **338**, 222–226 (2012). doi:10.1126/science.1224344 Medline

20. M. Jay, C. Haselgrove, D. Hamilton, J. D. Hill, J. Dent, Chariots and context: New radiocarbon dates from Wetwang and the chronology of iron age burials and brooches in east Yorkshire. *Oxf. J. Archaeol.* **31**, 161–189 (2012). doi:10.1111/j.1468-0092.2012.00384.x

21. A. Bricking, A. Hayes, R. Madgwick, An interim report on histological analysis of human bones from Fishmonger's Swallet, Gloucestershire. *Proc. Univ. Bristol Speleological Soc.* **29**, 67–86 (2022).

22. A. Hicks, *Medieval Town and Augustinian Friary: Settlement c1325–1700* (Canterbury Archaeological Trust, 2015).

23. Materials and methods are available as supplementary materials.

24. K. Cootes, J. Axworthy, M. Borrini, R. Carlin, J. Irish, D. Jordan, M. King, H. Russ, R. Swallow, M. Thomas, S. Valoriani, Father T. Williams, F. Petchey, Poulton, Cheshire: The investigation of a rural chapel in an evolving medieval landscape. *Church Archaeology* **23**, 43–53 (2023). doi:10.3828/churcharch.2023.23.43

25. P. Brotherton, P. Endicott, J. J. Sanchez, M. Beaumont, R. Barnett, J. Austin, A. Cooper, Novel high-resolution characterization of ancient DNA reveals C > U-type base modification events as the sole cause of post mortem miscoding lesions. *Nucleic Acids Res.* **35**, 5717–5728 (2007). doi:10.1093/nar/gkm588 Medline

26. A. W. Briggs, U. Stenzel, P. L. F. Johnson, R. E. Green, J. Kelso, K. Prüfer, M. Meyer, J. Krause, M. T. Ronan, M. Lachmann, S. Pääbo, Patterns of damage in genomic DNA sequences from a Neandertal. *Proc. Natl. Acad. Sci. U.S.A.* **104**, 14616–14621 (2007). doi:10.1073/pnas.0704665104 Medline

27. F. M. Key, C. Posth, J. Krause, A. Herbig, K. I. Bos, Mining metagenomic data sets for

ancient DNA: Recommended protocols for authentication. *Trends Genet.* **33**, 508–520 (2017). doi:10.1016/j.tig.2017.05.005 Medline

28. G. Tonkin-Hill, N. MacAlasdair, C. Ruis, A. Weimann, G. Horesh, J. A. Lees, R. A. Gladstone, S. Lo, C. Beaudoin, R. A. Floto, S. D. W. Frost, J. Corander, S. D. Bentley, J. Parkhill, Producing polished prokaryotic pangenomes with the Panaroo pipeline. *Genome Biol.* **21**, 180 (2020). doi:10.1186/s13059-020-02090-4 Medline

29. X. Didelot, D. J. Wilson, ClonalFrameML: Efficient inference of recombination in whole bacterial genomes. *PLOS Comput. Biol.* **11**, e1004041 (2015). doi:10.1371/journal.pcbi.1004041 Medline

30. X. Didelot, N. J. Croucher, S. D. Bentley, S. R. Harris, D. J. Wilson, Bayesian inference of ancestral dates on bacterial phylogenetic trees. *Nucleic Acids Res.* **46**, e134 (2018). doi:10.1093/nar/gky783 Medline

31. R. Bouckaert, T. G. Vaughan, J. Barido-Sottani, S. Duchêne, M. Fourment, A. Gavryushkina, J. Heled, G. Jones, D. Kühnert, N. De Maio, M. Matschiner, F. K. Mendes, N. F. Müller, H. A. Ogilvie, L. du Plessis, A. Popinga, A. Rambaut, D. Rasmussen, I. Siveroni, M. A. Suchard, C.-H. Wu, D. Xie, C. Zhang, T. Stadler, A. J. Drummond, BEAST 2.5: An advanced software platform for Bayesian evolutionary analysis. *PLOS Comput. Biol.* **15**, e1006650 (2019). doi:10.1371/journal.pcbi.1006650 Medline

32. M. Sikora, E. Canteri, A. Fernandez-Guerra, N. Oskolkov, R. Ågren, L. Hansson, E. K. Irving-Pease, B. Mühlemann, S. H. Nielsen, G. Scorrano, M. E. Allentoft, F. V. Seersholm, H. Schroeder, C. Gaunitz, J. Stenderup, L. Vinner, T. C. Jones, B. Nystedt, J. Parkhill, L. Fugger, F. Racimo, K. Kristiansen, A. K. N. Iversen, E. Willerslev, The landscape of ancient human pathogens in Eurasia from the Stone Age to historical times. bioRxiv 2023.10.06.561165 (2023); https://doi.org/10.1101/2023.10.06.561165.

33. Y. Takahashi, S. J. Cutler, M. Fukunaga, Size conversion of a linear plasmid in the relapsing fever agent *Borrelia duttonii*. *Microbiol. Immunol.* **44**, 1071–1074 (2000). doi:10.1111/j.1348-0421.2000.tb02605.x Medline

34. H. Murray, J. Errington, Dynamic control of the DNA replication initiation protein DnaA by Soj/ParA. *Cell* **135**, 74–84 (2008). doi:10.1016/j.cell.2008.07.044 Medline

35. C. Vink, G. Rudenko, H. S. Seifert, Microbial antigenic variation mediated by homologous DNA recombination. *FEMS Microbiol. Rev.* **36**, 917–948 (2012). doi:10.1111/j.1574-6976.2011.00321.x Medline

36. F. Röttgerding, P. Kraiczy, Immune evasion strategies of relapsing fever spirochetes. *Front. Immunol.* **11**, 1560 (2020). doi:10.3389/fimmu.2020.01560 Medline

37. R. D. Gilmore, B. A. Armstrong, K. S. Brandt, T. J. Van Gundy, A. Hojgaard, J. E. Lopez, A. R. Kneubehl, Analysis of variable major protein antigenic variation in the relapsing fever spirochete, *Borrelia miyamotoi*, in response to polyclonal antibody selection pressure. *PLOS ONE* **18**, e0281942 (2023). doi:10.1371/journal.pone.0281942 Medline

38. J. R. Fischer, K. T. LeBlanc, J. M. Leong, Fibronectin binding protein BBK32 of the Lyme disease spirochete promotes bacterial attachment to glycosaminoglycans. *Infect. Immun.* **74**, 435–441 (2006). doi:10.1128/IAI.74.1.435-441.2006 Medline

39. X.-G. Wang, J. M. Kidder, J. P. Scagliotti, M. S. Klempner, R. Noring, L. T. Hu, Analysis of differences in the functional properties of the substrate binding proteins of the *Borrelia burgdorferi* oligopeptide permease (Opp) operon. *J. Bacteriol.* **186**, 51–60 (2004). doi:10.1128/JB.186.1.51-60.2004 Medline

40. European Centre for Disease Prevention and Control (ECDC), Rapid Risk Assessment: Louse-borne relapsing fever in the EU, 19 November 2015 (2015); https://www.ecdc.europa.eu/en/publications-data/rapid-risk-assessment-louse-borne-relapsing-fever-eu-19-november-2015.

41. N. Rascovan, K.-G. Sjögren, K. Kristiansen, R. Nielsen, E. Willerslev, C. Desnues, S. Rasmussen, Emergence and spread of basal lineages of *Yersinia pestis* during the Neolithic decline. *Cell* **176**, 295–305.e10 (2019). doi:10.1016/j.cell.2018.11.005 Medline

42. K. Kristiansen, M. L. S. Sørensen, "Wool in the bronze age: Concluding reflections" in *The Textile Revolution in Bronze Age Europe*, S. Sabatini, S. Bergerbrant, Eds. (Cambridge Univ. Press, 2019), pp. 317–332.

43. L. C. Viñas-Caron, M. Nørtoft, P. Flemestad, J. Holm Jæger, C. Margariti, "From fleece to thread. Interdisciplinary evidence for the origins of sheep wool" in *The Common Thread: Collected Essays in Honour of Eva Andersson Strand*, U. Mannering, M.-L. Nosch, A. Drewsen, Eds. (Brepols Publishers, 2024), pp. 33–60.

44. K. M. Frei, U. Mannering, K. Kristiansen, M. E. Allentoft, A. S. Wilson, I. Skals, S. Tridico, M. L. Nosch, E. Willerslev, L. Clarke, R. Frei, Tracing the dynamic life story of a Bronze Age female. *Sci. Rep.* **5**, 10431 (2015). doi:10.1038/srep10431 Medline

45. P. A. Buxton, The control of lice. *BMJ* **2**, 603–604 (1940). doi:10.1136/bmj.2.4165.603 Medline

46. W. Li, G. Ortiz, P.-E. Fournier, G. Gimenez, D. L. Reed, B. Pittendrigh, D. Raoult, Genotyping of human lice suggests multiple emergencies of body lice from local head louse populations. *PLOS Negl. Trop. Dis.* **4**, e641 (2010). doi:10.1371/journal.pntd.0000641 Medline

47. N. Amanzougaghene, F. Fenollar, D. Raoult, O. Mediannikov, Where are we with human lice? A review of the current state of knowledge. *Front. Cell. Infect. Microbiol.* **9**, 474 (2020). doi:10.3389/fcimb.2019.00474 Medline

48. M. E. Allentoft, M. Sikora, K.-G. Sjögren, S. Rasmussen, M. Rasmussen, J. Stenderup, P. B. Damgaard, H. Schroeder, T. Ahlström, L. Vinner, A.-S. Malaspinas, A. Margaryan, T. Higham, D. Chivall, N. Lynnerup, L. Harvig, J. Baron, P. Della Casa, P. Dąbrowski, P. R. Duffy, A. V. Ebel, A. Epimakhov, K. Frei, M. Furmanek, T. Gralak, A. Gromov, S. Gronkiewicz, G. Grupe, T. Hajdu, R. Jarysz, V. Khartanovich, A. Khokhlov, V. Kiss, J. Kolář, A. Kriiska, I. Lasak, C. Longhi, G. McGlynn, A. Merkevicius, I. Merkyte, M. Metspalu, R. Mkrtchyan, V. Moiseyev, L. Paja, G. Pálfi, D. Pokutta, Ł. Pospieszny, T. D. Price, L. Saag, M. Sablin, N. Shishlina, V. Smrčka, V. I. Soenov, V. Szeverényi, G. Tóth, S. V. Trifanova, L. Varul, M. Vicze, L. Yepiskoposyan, V. Zhitenev, L. Orlando, T. Sicheritz-Pontén, S. Brunak, R. Nielsen, K. Kristiansen, E. Willerslev, Population genomics of Bronze Age Eurasia. *Nature* **522**, 167–172 (2015). doi:10.1038/nature14507 Medline

49. S. J. Cutler, E. M. Bonilla, R. J. Singh, Population structure of East African relapsing fever *Borrelia* spp. *Emerg. Infect. Dis.* **16**, 1076–1080 (2010). doi:10.3201/eid1607.091085 Medline

50. I. Matic, Mutation rate heterogeneity increases odds of survival in unpredictable environments. *Mol. Cell* **75**, 421–425 (2019). doi:10.1016/j.molcel.2019.06.029 Medline

51. G. G. R. Murray, A. J. Balmer, J. Herbert, N. F. Hadjirin, C. L. Kemp, M. Matuszewska, S. Bruchmann, A. S. M. M. Hossain, M. Gottschalk, A. W. Tucker, E. Miller, L. A. Weinert, Mutation rate dynamics reflect ecological change in an emerging zoonotic pathogen. *PLOS Genet.* **17**, e1009864 (2021). doi:10.1371/journal.pgen.1009864 Medline

52. D. Grimm, A. F. Elias, K. Tilly, P. A. Rosa, Plasmid stability during in vitro propagation of *Borrelia burgdorferi* assessed at a clonal level. *Infect. Immun.* **71**, 3138–3145 (2003). doi:10.1128/IAI.71.6.3138-3145.2003 Medline

53. W. R. Zückert, J. Meyer, Circular and linear plasmids of Lyme disease spirochetes have extensive homology: Characterization of a repeated DNA element. *J. Bacteriol.* **178**, 2287–2298 (1996). doi:10.1128/jb.178.8.2287-2298.1996 Medline

54. S. J. Cutler, I. J. Rinky, E. M. Bonilla, Does RecA have a role in *Borrelia recurrentis*? *Clin. Microbiol. Infect.* **17**, 195–197 (2011). doi:10.1111/j.1469-0691.2010.03249.x Medline

55. T. Meri, S. J. Cutler, A. M. Blom, S. Meri, T. S. Jokiranta, Relapsing fever spirochetes *Borrelia recurrentis* and *B. duttonii* acquire complement regulators C4b-binding protein and factor H. *Infect. Immun.* **74**, 4157–4163 (2006). doi:10.1128/IAI.00007-06 Medline

56. D. E. Wood, J. Lu, B. Langmead, Improved metagenomic analysis with Kraken 2. *Genome Biol.* **20**, 257 (2019). doi:10.1186/s13059-019-1891-0 Medline

57. I. Armit, The Wetwang/Garton Slack Project (WGSP) [data-set]. Archaeology Data Service (2015); https://doi.org/10.5284/1030285.

58. J. S. Dent, *The Iron Age in East Yorkshire*, British Archaeological Reports British Series (BAR Publishing, 2019.

59. D. Hardwick, Fishmonger’s Swallet, near Alveston, Gloucestershire: Description and history. *Proc. Univ. Bristol Spelaeological Soc.* **29**, 7–18 (2022).

60. M. Horton, Archaeology and television at Fishmonger’s Swallet. *Proc. Univ. Bristol Spelaeological Soc.* **29**, 23–28 (2022).

61. M. Cox, L. Loe, The human skeletal remains from Fishmonger’s Swallet, Alveston, Gloucestershire: Evidence for anthropogenic modification. *Proc. Univ. Bristol Spelaeological Soc.* **29**, 33–66 (2022).

62. A. Bricking, J. Peto, M. Horton, G. Mullan, Fishmonger’s Swallet, Alveston, Gloucestershire, radiocarbon dating. *Proc. Univ. Bristol Spelaeological Soc.* **29**, 29–32 (2022).

63. M. M. Emery, D. J. L. Gibbins, K. J. Matthews, *The Archaeology of an Ecclesiastical Landscape: Chapel House Farm, Poulton (Cheshire) 1995* (Chester City Council, Department of Development and Leisure Services, 1996).

64. J. Dabney, M. Knapp, I. Glocke, M.-T. Gansauge, A. Weihmann, B. Nickel, C. Valdiosera,

N. García, S. Pääbo, J.-L. Arsuaga, M. Meyer, Complete mitochondrial genome sequence of a Middle Pleistocene cave bear reconstructed from ultrashort DNA fragments. *Proc. Natl. Acad. Sci. U.S.A.* **110**, 15758–15763 (2013). doi:10.1073/pnas.1314445110 Medline

65. N. Rohland, I. Glocke, A. Aximu-Petri, M. Meyer, Extraction of highly degraded DNA from ancient bones, teeth and sediments for high-throughput sequencing. *Nat. Protoc.* **13**, 2447–2461 (2018). doi:10.1038/s41596-018-0050-5 Medline

66. M.-T. Gansauge, A. Aximu-Petri, S. Nagel, M. Meyer, Manual and automated preparation of single-stranded DNA libraries for the sequencing of DNA from ancient biological remains and other sources of highly degraded DNA. *Nat. Protoc.* **15**, 2279–2300 (2020). doi:10.1038/s41596-020-0338-0 Medline

67. M. Kircher, S. Sawyer, M. Meyer, Double indexing overcomes inaccuracies in multiplex sequencing on the Illumina platform. *Nucleic Acids Res.* **40**, gkr771 (2011). doi:10.1093/nar/gkr771 Medline

68. J. A. Fellows Yates, T. C. Lamnidis, M. Borry, A. Andrades Valtueña, Z. Fagernäs, S. Clayton, M. U. Garcia, J. Neukamm, A. Peltzer, Reproducible, portable, and efficient ancient genome reconstruction with nf-core/eager. *PeerJ* **9**, e10947 (2021). doi:10.7717/peerj.10947 Medline

69. M. Schubert, S. Lindgreen, L. Orlando, AdapterRemoval v2: Rapid adapter trimming, identification, and read merging. *BMC Res. Notes* **9**, 88 (2016). doi:10.1186/s13104-016-1900-2 Medline

70. H. Li, R. Durbin, Fast and accurate short read alignment with Burrows-Wheeler transform. *Bioinformatics* **25**, 1754–1760 (2009). doi:10.1093/bioinformatics/btp324 Medline

71. M. Poullet, L. Orlando, Assessing DNA sequence alignment methods for characterizing ancient genomes and methylomes. *Front. Ecol. Evol.* **8**, 105 (2020). doi:10.3389/fevo.2020.00105

72. J. Neukamm, A. Peltzer, K. Nieselt, DamageProfiler: Fast damage pattern calculation for ancient DNA. *Bioinformatics* **37**, 3652–3653 (2021). doi:10.1093/bioinformatics/btab190 Medline

73. H. Li, B. Handsaker, A. Wysoker, T. Fennell, J. Ruan, N. Homer, G. Marth, G. Abecasis, R. Durbin; 1000 Genome Project Data Processing Subgroup, The sequence alignment/map format and SAMtools. *Bioinformatics* **25**, 2078–2079 (2009). doi:10.1093/bioinformatics/btp352 Medline

74. W. Shen, S. Le, Y. Li, F. Hu, SeqKit: A cross-platform and ultrafast toolkit for FASTA/Q file manipulation. *PLOS ONE* **11**, e0163962 (2016). doi:10.1371/journal.pone.0163962 Medline

75. H. Li, htsbox (Github); https://github.com/lh3/htsbox.

76. S. Kalyaanamoorthy, B. Q. Minh, T. K. F. Wong, A. von Haeseler, L. S. Jermiin, ModelFinder: Fast model selection for accurate phylogenetic estimates. *Nat. Methods* **14**, 587–589 (2017). doi:10.1038/nmeth.4285 Medline

77. A. Rambaut, FigTree v1.3.1: Tree Figure Drawing Tool (2009); http://tree.bio.ed.ac.uk/software/figtree/ [accessed 2 November 2011].

78. R. R. Bouckaert, A. J. Drummond, bModelTest: Bayesian phylogenetic site model averaging and model comparison. *BMC Evol. Biol.* **17**, 42 (2017). doi:10.1186/s12862-017-0890-6 Medline

79. A. J. Page, B. Taylor, A. J. Delaney, J. Soares, T. Seemann, J. A. Keane, S. R. Harris, SNP-sites: Rapid efficient extraction of SNPs from multi-FASTA alignments. *Microb. Genom.* **2**, e000056 (2016). doi:10.1099/mgen.0.000056 Medline

80. T. Seemann, Prokka: Rapid prokaryotic genome annotation. *Bioinformatics* **30**, 2068–2069 (2014). doi:10.1093/bioinformatics/btu153 Medline

81. E. Paradis, K. Schliep, ape 5.0: An environment for modern phylogenetics and evolutionary analyses in R. *Bioinformatics* **35**, 526–528 (2019). doi:10.1093/bioinformatics/bty633 Medline

82. P. Jones, D. Binns, H.-Y. Chang, M. Fraser, W. Li, C. McAnulla, H. McWilliam, J. Maslen, A. Mitchell, G. Nuka, S. Pesseat, A. F. Quinn, A. Sangrador-Vegas, M. Scheremetjew, S.-Y. Yong, R. Lopez, S. Hunter, InterProScan 5: Genome-scale protein function classification. *Bioinformatics* **30**, 1236–1240 (2014). doi:10.1093/bioinformatics/btu031 Medline

83. R. C. Edgar, MUSCLE: Multiple sequence alignment with high accuracy and high throughput. *Nucleic Acids Res.* **32**, 1792–1797 (2004). doi:10.1093/nar/gkh340 Medline

84. A. R. Quinlan, I. M. Hall, BEDTools: A flexible suite of utilities for comparing genomic features. *Bioinformatics* **26**, 841–842 (2010). doi:10.1093/bioinformatics/btq033 Medline
